# Virtual Surgery and CFD in Rhinology: A Scoping Review

**DOI:** 10.3390/jcm15208069

**Published:** 2026-10-17

**Authors:** Konrad Szydłowski, Paulina Nogal, Andrzej Skorek, Agnieszka Kasprzyk-Tryk, Dmitry Tretiakow

**Affiliations:** 1 Clinical Department of Otolaryngology, Academy of Applied Medical and Social Sciences, The Nicolaus Copernicus Hospital in Gdansk, Copernicus Healthcare Entity, Powstańców Warszawskich 1/2, 80-152 Gdansk, Poland; 2 Department of Otorhinolaryngology, Centralsjukhuset Karlstad, 651 85 Karlstad, Sweden

**Keywords:** machine learning, nasal patency, wall shear stress, mucosal cooling, patient-specific modeling, computer-assisted surgical planning, empty nose syndrome

## Abstract

**Background:** Nasal obstruction is one of the most common reasons for which patients present to an otolaryngologist, yet the outcomes of operations aimed at improving nasal patency remain inconsistent, and the correlation between conventional objective measures and perceived patency is uncertain. Computational fluid dynamics (CFD) allows nasal airflow to be simulated from craniofacial computed tomography; combined with software that reconstructs and modifies sinonasal anatomy, it permits a planned procedure to be reproduced digitally and its functional impact predicted before any tissue is removed. This scoping review maps how CFD-based virtual surgery has been used in rhinology, how the simulations were validated, and which clinical outcomes were reported. **Methods:** Following the PRISMA extension for scoping reviews, PubMed and Scopus were searched in October 2025 for studies published between 2015 and 2025. Inclusion criteria restricted papers to English-language studies of virtual surgery tools involving CFD in humans or human-derived models. Pediatric (<18 years), sleep apnea, and orthognathic studies were excluded, as were studies of other parts of the respiratory tract and environmental or toxicological work. Duplicates were removed in Mendeley, and two authors independently screened titles and abstracts. Data were charted by two authors. The review was not registered. **Results:** Forty-one studies were included, covering the nasal septum, the inferior and middle turbinates, the ostiomeatal complex, the maxillary and sphenoid sinuses, septal perforations, and postoperative synechiae. Virtual results were compared with actual postoperative anatomy, airflow, or symptoms in only 7 of the 41 studies and agreed with them in most respects. In several small studies, a greater extent of resection did not improve airflow further and reduced mucosal heat and moisture exchange, wall shear stress, and regional cooling, but the extent that appeared best depended on the anatomy and the outcome considered. These parameters have been proposed as better correlates of the subjective sense of patency than resistance alone; this has rarely been tested in patients. The most recent work applies machine learning and adjoint optimization as research tools for planning support. **Conclusions:** CFD-based virtual surgery is a promising tool for planning the extent of rhinological procedures. Its routine use will require standardized methods, prospective validation, and correlation with patient-reported outcomes (NOSE, SNOT-22); larger studies with such endpoints are still lacking.

## 1. Introduction

Nasal obstruction is one of the most common reasons patients present to an otolaryngologist, and operations aimed at improving nasal patency are among the most frequently performed procedures in otolaryngology departments. Despite the fairly obvious causes of nasal obstruction—such as a deviated nasal septum, hypertrophy of the nasal turbinates, or chronic rhinosinusitis—and despite well-standardized surgical procedures, the outcomes of these operations remain variable. The computational fluid dynamics (CFD) method, originally developed for the aerospace industry, has in recent years been applied to many areas of medicine. In otolaryngology, it has been used to simulate nasal airflow based on imaging studies, usually craniofacial computed tomography. Combined with methods that allow the anatomy of the nasal cavity and paranasal sinuses to be reconstructed and modified, this has given us the ability to simulate surgical procedures and to assess them by calculating parameters related to nasal airflow [1,2]. Given the inconsistent and unsatisfactory outcomes of rhinological procedures, such as septoplasty, it seems reasonable to seek options that offer a greater likelihood of a favorable clinical outcome. Two randomized controlled trials have shown that septoplasty improves nasal obstruction more than non-surgical management—the pragmatic trial of van Egmond et al. [3] and the multicenter NAIROS trial [4]—and a recent meta-analysis points in the same direction [5]. However, these benefits are not consistent, reproducible, or long-lasting in all patients, as shown in a Swedish registry of more than 11,000 septoplasties [6]. The situation is similar for procedures involving the nasal turbinates [7] and the sinuses (FESS). The explanation for this lies in the anatomy and physiology of the nose and paranasal sinuses, whose variability across the population poses a challenge for the rhinosurgeon. Beyond the standard quantitative approach to nasal airflow, the systematic review by André et al. [8] found that the correlation between rhinomanometry or acoustic rhinometry and perceived nasal patency remains uncertain. This is consistent with earlier work [9,10]. Even the severity of an anatomical septal deviation does not reliably predict the degree of subjective obstruction [11]. In recent years, increasing attention has been paid to qualitative parameters such as wall shear stress and the cooling capacity of the nasal cavity, and the role of the receptors that sense air movement and are responsible for the patient’s central perception of nasal patency is being increasingly recognized. Because of these complex relationships, surgeons need methods to objectify the expected outcomes, as well as tools that would help in planning procedures. This is where virtual (in silico) surgery methods come in: the preoperative anatomical model is digitally edited to simulate a planned procedure, and CFD is used to predict its functional impact before any tissue is removed [12]. In principle, this allows the extent and location of surgery to be tailored to the individual patient and the likely outcome to be estimated in advance and facilitates the avoidance of unnecessary tissue removal. A recent narrative review [12] summarized virtual septoplasty in nasal airway obstruction. Our review is broader: it covers surgery of the septum, turbinates, paranasal sinuses, septal perforations and synechiae and compares the computational methods used in the included studies. Given the growing number of studies on this subject, as well as the rapid development of artificial intelligence and machine learning methods, we decided to summarize the progress made in the past decade and to examine the further possibilities of this approach. We started the search in 2015 because this covers the last ten years and because more relevant studies were published from 2015 onwards than before. We chose a scoping review because the literature consists mostly of small, heterogeneous simulation studies that cannot be pooled. The review addresses four questions: (1) Which rhinological procedures and pathological changes have been modeled with CFD-based virtual surgery? (2) Which computational methods were used? (3) How were the simulations validated? (4) Which clinical outcomes were reported?

## 2. Materials and Methods

This scoping review was conducted following the Preferred Reporting Items for Systematic Reviews and Meta-Analyses (PRISMA) extension for scoping reviews (PRISMA-ScR) [13]. The completed checklist is provided in Table S3. The research questions, eligibility criteria and search strategy were agreed upon by the authors before the search. This was done informally, and no written protocol was registered or published. PROSPERO does not accept scoping reviews, and we did not use another registry.

### 2.1. Search Strategy

The search was conducted using the PubMed and Scopus databases. The last search was run in October 2025 in both databases. Articles published between 2015 and 2025 were included in the review. The search terms used in PubMed were (“computational fluid dynamics”[Title/Abstract] OR “CFD”[Title/Abstract] OR “numerical simulation”[Title/Abstract] OR “airflow simulation”[Title/Abstract] OR “flow simulation”[Title/Abstract] OR “computational modeling”[Title/Abstract] OR “computational modelling”[Title/Abstract] OR “numerical model”[Title/Abstract]) AND (nasal OR nose OR sinonasal OR “nasal cavity” OR “nasal airway” OR “nasal obstruction” OR “nasal septum” OR septal OR “paranasal sinus” OR “paranasal sinuses” OR turbinate OR turbinates OR rhinology OR rhinologic OR rhinosinusitis OR “nasal valve” OR “empty nose”) AND (surgery OR surgical OR virtual OR “surgical planning” OR “surgery planning” OR septoplasty OR turbinectomy OR turbinoplasty OR antrostomy OR maxillectomy OR FESS OR “sinus surgery” OR resection). In PubMed, the [Title/Abstract] tag was applied to the first block only, and the other two blocks were entered without field tags. The Scopus search used the same three concept blocks, each within the TITLE-ABS-KEY field. Both searches were limited to publication years 2015–2025 and to English-language records; the complete strings, as run, are given in Table S2. The reference lists of the included studies were not screened systematically, and no other supplementary search was performed. The search yielded 694 records (349 from PubMed, 345 from Scopus).

### 2.2. Inclusion and Exclusion Criteria

Studies on the use of Virtual Surgery Tools involving CFD were included. The search was restricted to articles written in English. Only human subjects/human-derived models with in vivo and/or in silico simulation studies were included in the review. The search excluded pediatric (<18 years), sleep apnea, and orthognathic studies, as well as research on areas extending to other parts of the respiratory tract apart from the nose and paranasal sinuses. We have also excluded environmental and toxicology studies. Only studies of adult subjects (18 years or older) were eligible; age was not charted for individual studies. Studies were eligible when a surgical intervention, or a pathological change relevant to surgery (septal deviation, septal perforation or synechiae), was created in a digital model of the nose or paranasal sinuses and its effect was assessed with CFD. We accepted three kinds of application: prospective planning of an operation that was then performed, retrospective comparison of a virtual with an actual postoperative state, and simulation of alternative resections or of created pathologies without a real operation. Eligible publications were full reports of original simulation studies published in journals or in conference proceedings indexed in PubMed or Scopus. Two studies at the edge of this scope were kept: the study of pollen deposition after middle turbinectomy (Section 3.6), because it simulates a consequence of a specific rhinological operation, unlike the excluded environmental studies, which involved no surgical intervention; and the study of sinus floor elevation (Section 3.3), because it models a procedure that changes the maxillary sinus and its ventilation, although the indication is dental.

### 2.3. Study Selection

After duplicates were removed using Mendeley’s reference management software v2.137.0, two authors (K.S. and P.N.) independently screened the titles and abstracts. Articles selected at this stage were assessed in full text by all authors. Disagreements were resolved by discussion with a third author (D.T.) when consensus could not be reached. The study selection process is presented in Figure 1. Four reports were excluded after full-text assessment; they are listed with the reasons in Table S1.

### 2.4. Data Charting and Synthesis

Data were charted independently by two authors (K.S. and P.N.), each in a separate spreadsheet, and their entries were then compared; disagreements and uncertain entries were resolved by a third author (D.T.). For each study, we recorded the number of patients or models, the anatomical region, the virtual intervention, and the main outcomes (Table 1), and the geometry source, software, flow regime, turbulence treatment, heat and humidity modeling, and validation approach (Table 2). Entries that the primary article did not report were marked “NR”. The funding sources of the included studies were not charted. Studies were grouped by anatomical region and type of virtual intervention (Section 3.2, Section 3.3, Section 3.4, Section 3.5, Section 3.6 and Section 3.7) and summarized narratively. Because the aim was to map the evidence, we did not appraise the methodological quality of the studies and did not perform a meta-analysis. All entries in Table 2 and the numbers quoted in the Results were checked against the full texts of the included studies.

## 3. Results

### 3.1. Characteristics of the Included Studies

The 41 included studies were published between 2015 and 2025: 23 in 2015–2020 and 18 in 2021–2025, with the highest annual numbers in 2018 (n = 7) and 2020 (n = 6). The methodological characteristics of each study are summarized in Table 2, and Table 3 shows how the studies are distributed by anatomical region and type of external comparison. Most reconstructions were built from a single patient’s computed tomography scan; only a minority were based on or collected from larger samples, the largest being 14 patients [48], followed by two cohorts of 10 [19,37]. One study used magnetic resonance imaging rather than computed tomography [32], and two used cone-beam computed tomography: one acquired for a dental indication [51] and one in healthy subjects [37]. Several groups compared their virtual results against a healthy reference cohort. Three studies from the Medical College of Wisconsin used the same set of 47 healthy adults as reference values [29], as the source of a benchmark healthy septum [35], and as the pool from which the 10 subjects of [37] were selected. The two studies by Siu et al. [34,39] analyzed the same 10 subjects (8 patients and 2 healthy controls). Such publications are therefore not independent samples.

A few research groups account for a large share of the literature. The Wisconsin group, working with the Garcia–Kimbell workflow, consistently segments in Mimics and solves in ANSYS Fluent under a steady, laminar assumption [14,19,29,35,37]; ICEM-CFD is named as the mesh generator in [29,35,37]. The flow was driven differently in these studies (15 L/min in [14,19], a fixed pressure drop of 15 Pa in [37], and other conditions in [29,35]), and heat and humidity were modeled in only some of them (both in [14,19], heat only in [29]). A similar sequence of segmentation, ICEM-CFD meshing, and steady laminar simulation in Fluent was used in [22,30,32,52]. A second set of studies [34,38,39,40,46,47,53] used 3D Slicer for segmentation and ANSYS Fluent for the simulations, most of them with steady laminar flow at 15 L/min (5 and 15 L/min in [40]). Heat and moisture exchange was modeled in four of these [38,39,46,47], and three of them [38,46,47] used a submucus wall model, in which the mucus and submucosal layer provide thermal resistance. These workflows differ in flow conditions and in the treatment of heat and humidity, so their results cannot be compared directly, even when they address the same anatomical question.

ANSYS Fluent was by far the most common solver, named in 28 of the 41 studies, but it was used in several different configurations, ranging from purely laminar, steady simulations to unsteady, turbulent ones. Beyond ANSYS Fluent, the review revealed a handful of heterogeneous sets of tools: the MeComLand and DigBody tools of the Spanish group [23,24,25,26] (OpenFOAM is named in [25]), OpenFOAM in [36,50], Star-CCM+ [33], Autodesk CFD [51], and a distinct current of lattice-Boltzmann solvers [21,28,42,49].

The treatment of turbulence was similarly varied. Twenty-six of the 41 studies (63%) assumed laminar flow throughout. The others used a Reynolds-averaged turbulence model (shear-stress-transport k–ω in four studies [20,27,50,51] and renormalization-group or low-Reynolds k–ε in two [31,41]), large-eddy simulation [28], a direct numerical simulation on a fifty-million-cell mesh [36], or lattice-Boltzmann solvers with no separate turbulence model stated [21,42,49], and one study did not state its flow model [17]. Three studies used a laminar assumption together with a turbulence model, for different flow rates or model components [43,53,54]. In some studies the choice followed the flow rate: [20,27] adopted a turbulence model for flow above about 25 L/min, citing earlier work, ref. [43] used a laminar assumption at 15 L/min and a transition model at 60 L/min, and [54] used a turbulence model above about 20 L/min. We could not identify a single rule, and the authors of [54] noted that there is no specific critical flow rate for the transition. Heat was modeled in 18 of the 41 studies and heat together with humidity in 11 of them, and the same laboratories often modeled heat and humidity in some papers and omitted them in particle-deposition studies. Where reported, the inspired air was set at 20 °C in 14 of the 18 studies (19 °C in [33], 25 °C in [45,48], and 12–40 °C in [39]); inspired relative humidity was 30–50% (13–80% in [39]), and the mucosal or wall temperature was 32.6–37 °C, with 100% relative humidity at the mucosa where humidity was modeled (Table 2).

The choice of turbulence model may affect the outputs that the reviewed studies relate to symptoms. In the included studies, its effect was shown for particle deposition, which increased at 60 L/min because of turbulent dispersion [43], and for the strongly fluctuating flow near the mucosa after medial maxillectomy, which was proposed as a cause of crusting [36]. Heat and humidity transport, in contrast, was in most studies calculated under a steady laminar assumption at 15 L/min [14,19,38,39,46,47]. We found no included study that compared laminar and turbulent solutions of these outputs at the same flow rate, so the size of the difference is not known. Future studies should state the flow rate, the turbulence model and the reason for choosing it, and should test how much heat flux, wall shear stress and humidity change with that choice.

Validation remained the weakest aspect of the field. Here, validation means a comparison of the simulated results with an independent external reference; mesh-independence testing checks the numerical solution but not whether it reflects the real airway. Seven of the 41 studies compared their simulations with an actual postoperative state: with postoperative imaging or with airflow computed from postoperative CT [21,28,33,35,52], or with symptoms after the real operation [23,26,33]. These comparisons involved one to seven patients each. Six studies compared their results with experimental measurements or with published simulations [15,27,41,43,44,54], and three compared them only with a healthy reference group [29,34,39], which gives normal values but cannot confirm a predicted surgical result. The remaining 25 studies reported no external comparison; in three of them the only check was a benchmark on a simple geometry [42], validation in the authors’ earlier work [48], or a review by ENT surgeons [50]. This lack of validation against clinical outcomes is discussed further in Section 4.5.

### 3.2. Nasal Septum and Inferior Turbinates

Most of the reviewed studies focused on virtual interventions involving the nasal septum and the inferior turbinates, as these are the most common clinical causes of nasal obstruction and impaired nasal breathing. Hariri et al. [14] analyzed sinus CT scans of five patients with turbinate hypertrophy in order to find the optimal extent of resection. Using virtual surgery, they compared inferior turbinoplasty performed along its lower aspect with turbinoplasty performed along its medial aspect. No differences in nasal resistance were found between the different extents of inferior turbinate correction; however, nasal air conditioning capacity was more affected by medial resections, which may translate into the humidification efficiency of the nose. Simmen et al. [17] simulated a pyriform turbinoplasty, in which bone of the frontal process of the maxilla and part of the lacrimal bone is removed under the mucosa, on a CT-based model of one patient with inferior turbinate hypertrophy, using an unsteady simulation of the breathing cycle. After the virtual procedure, the distance between the septum and the inferior turbinate increased by about 2 mm, and when the procedure was extended to lateralization of the lateral nasal wall, the airspeed in the inferior and middle meatus fell from 0.3 to 0.1 m/s, with little change in the airflow distribution in the posterior nasal cavity. The authors considered this more favorable than the changes reported after inferior turbinate resection, but this was a single model without resistance, flow rate, or heat exchange outcomes, and turbinate resection was not simulated. Burgos et al. [26], using the Dig Body tool to build 3D models and run CFD simulations, compared the effects of virtual surgery with actual postoperative outcomes. The study included two patients who underwent septoturbinoplasty and ESS combined with turbinoplasty, respectively. A virtual simulation of the potential surgical extent was performed first, and then the extent proposed by the virtual tools was actually carried out. Symptoms resolved in both patients, at three and six months respectively, but this was assessed qualitatively, without a symptom scale or postoperative measurements. In a further study, Burgos et al. [25] compared various rhinosurgical techniques in a model patient with a deviated septum (septoplasty, turbinectomy, spreader grafts, J-flap, and combinations among them). These were not the optimal techniques for this patient, but interestingly, mucotomy alone had an effect comparable to septoplasty in improving nasal patency in this simulation. This result comes from a single model and does not show that turbinoplasty can replace septoplasty in patients with a deviated septum.

Using CFD on 3D models built from the sinus CT scans of two patients with nasal obstruction, SanMiguel-Rojas et al. [23] ran simulations in search of the optimal extent of surgery on the nasal septum and the inferior turbinates. The rhinosurgeon, working together with engineers, performed a series of virtual septoplasties and turbinoplasties, looking for the surgical extent that was optimal in terms of CFD parameters. Once a satisfactory improvement in airflow parameters (flow rate, pressure drop) had been achieved based on the simulations, the actual surgical procedures were carried out. Postoperative outcomes were assessed with the SNOT-22 scale, with scores falling from 25 to 8 in the first patient and from 34 to 11 in the second. These are descriptive results in two patients without a control group.

Focusing exclusively on the inferior turbinates, Lee et al. [22] analyzed five patients, creating six different virtual methods and extents of inferior turbinate resection and their effect on nasal airflow. One of the most important observations was that the best results were obtained when the volume of the inferior turbinate was reduced along its entire length, compared with partial corrections. For every 1 mL of volume reduced in the inferior turbinate, total nasal airflow increased as follows: in patient 1 by 13.97 mL/s; in patient 2 by 6.07 mL/s; in patient 3 by 9.84 mL/s; in patient 4 by 25.9 mL/s; and in patient 5 by 24.27 mL/s.

The results of partial inferior turbinate resections were not reproducible, and, surprisingly, in some patients, posterior resections improved airflow parameters more than anterior resections. The results of partial resections differed even between the two sides in the same patient. In the study by Clipp et al. [21], virtual procedures on the nasal septum and inferior turbinates were performed in seven patients in collaboration with an experienced rhinosurgeon. The patients then underwent identical actual procedures designed on the basis of the virtual models. All patients had a postoperative sinus CT scan, which was then compared with the virtual models using 3D Slicer and CFD analysis. The authors reported satisfactory results, with the postoperative anatomy closely matching the virtual plan. To objectively assess how faithfully the actual surgery reproduced the virtual procedures, several landmark points within the septum were defined and analyzed across the pre-surgery CT, post-surgery CT, and virtual surgery scans. Across the seven cases, the average pre- to post-surgery distance was 1.2 mm, with a maximum of 4.75 mm, whereas the average virtual-surgery to post-surgery distance was 0.49 mm, with a maximum of 2.19 mm. This indicates that the surgeon was able to reproduce the virtual plan, which is not the same as predicting airflow or symptoms. Frank-Ito et al. [29] used the sinus CT scans of two patients with nasal obstruction to build seven virtual surgical models combining different extents of septoplasty and turbinoplasty. The results of the virtual interventions were compared with the airflow of control patients without nasal obstruction. In the first patient, the best effect was achieved with septoplasty alone, whereas the second patient obtained the best results with a combination of procedures (septoplasty plus inferior turbinoplasty). These results show that the optimal surgical extent can be difficult to determine, and they suggest that virtual simulation could help to tailor the extent of surgery to the individual patient and possibly reduce the invasiveness of the procedure.

The aim of the study by Berger et al. [28] was to identify SRPs (surgically relevant points), that is, locations within the nasal cavity whose correction could improve nasal airflow parameters. Sinus CT scans of one healthy patient and three patients with nasal obstruction were analyzed, and with CFD tools, the authors identified specific points along the airflow path at which a substantial drop in flow pressure occurred. These sites were then corrected virtually so as to reduce the pressure drop until the effect seen in the control patient was reached, that is, a pressure drop below 1 Pa/mm. The authors suggest that the tool may be useful in planning surgical procedures within the nasal cavities. An important limitation of this method is that the SRPs lie within structures that are not typically corrected surgically and that carry a risk of damage to vessels and nerves, for example, in the roof of the nasal cavity.

Li et al. [31] assessed the effect of resecting the posterior part of the nasal septum on nasal airflow and on impairment of its air-warming function. The aim of the study was to determine the maximum extent of resection that would not adversely affect the physical parameters of nasal airflow. A CT scan of one patient was analyzed, and three different virtual extents of posterior septal resection were performed, of 1, 2, and 3 cm, respectively. Compared with the control model, no marked differences were found in average and peak airflow velocity, average wall shear stress, airflow temperature, or turbulent kinetic energy, which may suggest that resection of up to 3 cm of the posterior nasal septum has little effect on the warming function of the nose and does not cause marked turbulent disturbances in this one-patient model.

Xiong et al. [32] compared the effects of different extents of virtual inferior turbinate resection in three patients with inferior turbinate hypertrophy. Based on MRI images, the side with greater obstruction (MOS) was compared with the side with lesser obstruction (LOS), and CFD simulations were used to assess the effect of the extent of turbinate resection on airflow resistance. It was shown that similar improvements in airflow can be obtained with procedures that are less invasive and cause less tissue damage. In patients 1 and 2, 35% and 56% smaller inferior turbinate resections, respectively, produced airflow improvements similar to those of a total turbinectomy. Sensitivity analysis indicated that leaving the narrowest segment untreated—even by just 3.4–7.8% less resected volume—led to a 22–32% drop in airflow through the obstructed side, emphasizing how critical that region is.

Radulesco et al. [33] assessed the accuracy of predicting septoplasty outcomes using virtual surgery and CFD. Using CFD, they compared the results of an actual endoscopic septoplasty (PS) with those of a virtual septoplasty (VS) in two patients with nasal obstruction. The study used imaging of two patients with a deviated septum. The patients had craniofacial CT scans taken before surgery and at least three months afterward; the postoperative imaging had been performed for reasons unrelated to the surgery. Two different surgeons performed the actual and the virtual procedures, and the comparison was retrospective. A satisfactory clinical outcome was achieved (the NOSE score in patient 1 fell from 70/100 before surgery to 0/100 after surgery; in patient 2, it fell from 90/100 before surgery to 0/100 after surgery). In addition, a strong correlation in airflow parameters was demonstrated between the actual-surgery and the virtual-surgery models—for heat flux (VS and PS respectively, r = 0.93, 95% CI 0.78–0.98), total pressure (r = 0.96, 95% CI 0.87–0.99), temperature (r = 0.98, 95% CI 0.95–0.99), and wall shear stress (r = 0.92, 95% CI 0.75–0.97) (all *p* < 0.01). These coefficients come from repeated measurements (several locations and time points of the breathing cycle) in the same two patients, so they are not independent observations, and a high correlation does not exclude a systematic difference between the models; the authors reported no differences between the virtual and actual models in the CFD measurements.

Siu et al. [34] used CFD to compare the effects of different extents of virtual inferior turbinate surgery—total inferior turbinectomy and inferior turbinoplasty. Eight patients with inferior turbinate hypertrophy were included, and the results were compared with those of a control group of two patients without nasal obstruction. Total turbinectomy resulted in lower wall shear stress (WSS) in the inferior nasal cavity than turbinoplasty and the control group (0.040 Pa vs. 0.054 Pa vs. 0.057 Pa), although resistance was slightly more reduced after turbinectomy. The authors interpreted the lower WSS as a sign of reduced mucosal cooling, citing earlier work; heat flux was not calculated in this study. Larger areas of mucosal cooling have been associated with better subjective nasal patency scores in previous studies, and the authors related reduced cooling to the disturbed stimulation of cooling receptors seen in patients with empty nose syndrome, who paradoxically sense nasal obstruction in the absence of any objective barrier to airflow. Neither symptoms nor receptor stimulation were assessed. Turbinoplasty resulted in ventilation, pressure, and wall shear stress profiles closer to those of healthy models. Both the ventilation and the WSS profiles of the turbinoplasty group more closely resembled those of the healthy group, which suggests that more extensive resection of inferior turbinate tissue did not bring additional benefit in these models and that total inferior turbinectomy may worsen airflow parameters that have been linked to the subjective sensation of nasal patency.

Moghaddam et al. [35] assessed the effects of virtual septoplasty in a single patient with a deviated septum, comparing benchmark CFD parameters derived from the CT images of 47 healthy control patients. The virtual septoplasty was designed based on the preoperative CT scans, identifying the locations where airflow parameters deviated from the benchmarks. In this single patient, the virtual and the actual postoperative results, compared retrospectively, were close for both the minimal airspace cross-sectional area (0.05 cm^2^ pre-surgery, 0.54 cm^2^ virtual surgery, 0.50 cm^2^ actual surgery) and nasal resistance (0.91 Pa·s/mL pre-surgery, 0.08 Pa·s/mL virtual surgery, 0.08 Pa·s/mL actual surgery).

In a subsequent study, Siu et al. [39] again assessed the difference between two methods of inferior turbinate surgery—total inferior turbinectomy and inferior turbinoplasty—this time evaluating the effects of the surgery on nasal heat exchange and humidification. Virtual surgery was performed in eight patients with inferior turbinate hypertrophy, and the effects of these interventions were compared under three different, virtually adjusted environments: ambient air, cold, dry air, and hot, humid air. In ambient air and cold, dry air, total heat flux was highest before surgery, intermediate after turbinoplasty, and lowest after turbinectomy (all *p* < 0.001). The pairwise differences printed in the source for hot, humid air are internally inconsistent (for example, a negative difference of −0.24 W for the pre-operative versus turbinoplasty comparison), and some of the differences do not add up, so we report only the direction and significance of the changes. Nasal conditioning is noticeably altered after inferior turbinate reduction surgery, especially with turbinectomy, when exposed to cold, dry inspired air. The degree of impairment is small across the simulated range of environmental conditions (T = 12–40 °C; RH = 13–80%). Regardless of the type of surgery, reduced air–mucosal heat exchange and a lower moisture-carrying capacity at cooler temperatures, together with disturbed airflow distribution, may have a desiccating effect and lead to crusting in the nasal cavity.

In the study by Campbell et al. [37], the authors assessed the effect of different extents and locations of virtually created septal deviations on airflow parameters. Ten healthy subjects were included, in whom the nasal cavity was reconstructed from CT imaging. A CFD airflow simulation was then run for each patient for four different extents of virtually created septal deviation. Parameters such as the mCSA (minimal cross-sectional area) and resistance were assessed. The mean (±SD) mCSA in the left cavity was 0.80 ± 0.18 cm^2^ in the healthy models. As the virtually created septal deviation increased, the mCSA fell to 0.64 ± 0.14, 0.38 ± 0.10, 0.19 ± 0.07, and 0.09 ± 0.06 cm^2^, respectively. The authors observed a strong correlation between the positive results after septoplasty and the average CSA of the post-valve airway (r = 0.937), with a wider post-valve CSA leading to better flow increases after septoplasty. They found a strong correlation between the minimum cross-sectional area (mCSA) of the nasal airway and nasal airflow, but this correlation was significant only when the mCSA was below 0.20 cm^2^, indicating a very severe septal deviation. When the mCSA exceeds 0.20 cm^2^, a wider post-valve airway on the same side as the deviation is associated with a better airflow response after septoplasty. In cases where the ipsilateral posterior nasal cavity is wide, anterior deviations have a disproportionately larger effect on nasal resistance. Therefore, increases in mCSA above 0.20 cm^2^ were associated with larger and more predictable improvements in airflow in the simulations. In contrast, when the post-valve airway is narrow, the resistance is more evenly distributed throughout the nasal airway, and in such cases, correcting an anterior septal deviation may not be enough to restore normal airflow. The authors conclude that surgeons should carefully evaluate the ipsilateral nasal airway located behind the septal deviation, and that an unrecognized small post-valve CSA may explain a persistent sensation of nasal obstruction after septoplasty. These are hypotheses derived from simulated deviations in healthy subjects. The authors suggest that inferior turbinate reduction on the same side as the septal deviation may magnify airflow after the procedure in patients with a narrow post-valve airway.

Waldmann et al. [42], using CFD on a patient’s CT scan with a deviated septum, performed a virtual septoplasty. An advanced analysis of nasal airflow parameters was carried out, along with the effect on the nasal cavity’s air-warming capacity, and the results were referred to normal values for healthy patients drawn from the literature. The results indicate that the surgery has minimal impact on the nasal cavity’s heating capacity; however, the overall flow resistance is reduced by 25.3%. Correcting the septal deviation also helps to redistribute the mass flux. Monitoring pressure loss and temperature changes during surgery suggests that complete tissue removal is not necessary to achieve improved conditions. The authors are now integrating computational fluid dynamics (CFD) software with a machine-learning algorithm that optimizes the geometry of the nasal cavity in areas designated by the surgeon, based on respiratory fluid mechanics. Once trained, this system can predict surgical solutions much faster than detailed numerical simulations.

The study by Tao et al. [43] also assessed the effect of a virtual septoplasty on nasal airflow, with particle deposition as an additional parameter under evaluation. They used CFD based on the CT scan of a single patient with a deviated septum. Two different septoplasty simulations were prepared, VS1 and VS2; the second of these assumed, in addition to correction of the cartilaginous and bony parts of the septum, correction of the nasal vestibule as well, with an emphasis on plasty of the caudal part of the quadrangular cartilage. The surgical extent in the VS2 model gave the best results in terms of airflow and airflow allocation. The baseline flow rate (L/min) on the side of the deviation was 2.4 L/min, and the airflow allocation was 16%, whereas in the virtual models after septoplasty, these were 3.6 L/min and 24% for VS1 and 5.0 L/min and 33.3% for VS2. The results show that, despite correction of the deviation, the parameters did not even out between the two sides, which the authors attribute to the influence of the inferior turbinates and the nasal cycle. The authors suggest using CFD analysis to optimize the extent of the procedure, and the results draw attention to the role of the anterior portion of the septal deviation in nasal airflow, which may be key to achieving satisfactory surgical outcomes.

The use of a reinforcement learning algorithm to predict and design adequate surgical outcomes was proposed by Rüttgers et al. [49]. The authors used CFD simulations based on preoperative CT scans from two patients—one with a deviated septum and the other with enlarged inferior turbinates—and combined them with machine-learning techniques. A novel RL algorithm was developed to predict optimal surgical outcomes from data derived from CFD simulations. Emphasis was placed on two parameters when judging the optimal surgical extent: nasal flow, expressed as the pressure difference between the nostrils and the pharynx (1), and the heating of incoming air (2). Several evaluations were performed using factors of different importance for the two criteria, pressure loss and temperature increase. The optimal surgical extent was different depending on which parameter was emphasized. For the patient with a deviated septum, assuming equal weights for the two parameters, the algorithm suggested a less invasive septal correction than the surgeon’s plan; with greater weight on nasal airflow, the approach involved a more robust septal correction, contrary to the plan, which had favored the heating capability. For the patient with inferior turbinate hypertrophy, with equal weights on the two criteria, the suggested plan included a nearly complete inferior turbinectomy and a moderate reduction of the middle turbinate; an increased weight on nasal airflow led to an additional reduction of the middle turbinate, while a greater weight on the second parameter yielded a solution with only slight reductions in both turbinates, focusing on adequate heat exchange. As can be seen, the two criteria considered in the study stand somewhat in opposition to each other. Neither criterion on its own provides information about the optimal surgical extent, but when analyzed together, they can offer an important suggestion for the surgeon planning the procedure.

The aim of the study by Macellari et al. [50] was to develop a CFD-based tool to help surgeons plan septoplasty. The study used the CT scans of three patients with a deviated septum and subjective nasal obstruction. Adjoint-based optimization was used to create a map of surface-sensitivity points indicating the areas of the nasal cavity that require correction to improve nasal patency. Virtual septoplasties were performed at the sites indicated by the algorithm, and airflow parameters were compared between the pre- and postoperative states. The results show lower nasal resistance after the virtual septoplasty in all three patients (in Pa·s/mL: P1, 0.091 before vs. 0.081 after; P2, 0.360 vs. 0.311; P3, 0.037 vs. 0.033), with no marked difference in the percentage distribution of airflow between the two sides; no statistical test was applied.

The subject of the study by Odeh et al. [52] was an analysis of the effect of total inferior turbinate removal on airflow parameters and a comparison of these results with the values of patients suffering from empty nose syndrome. The authors retrospectively recruited six patients with nasal obstruction who had undergone turbinate reduction surgery and had a repeat CT scan. After the actual operation, the NOSE score fell from 72.5 ± 13.2 to 10.8 ± 9.8 and the unilateral visual analog scale score of nasal obstruction from 6 ± 2.56 to 1.2 ± 1 (both *p* < 0.001); these scores describe the outcome of the actual surgery, not of the virtual total turbinectomy. A total inferior turbinectomy was simulated on the preoperative CT scans, and the CFD results of the virtual turbinectomy were compared with those of the actual postoperative CT scans; these parameters were additionally compared with those of 27 patients with empty nose syndrome from the authors’ earlier studies. Nasal resistance fell from 0.21 Pa·s/mL at baseline to 0.12 after the actual surgery (*p* = 0.06) and to 0.10 after the virtual turbinectomy (*p* = 0.01); the values after the actual surgery, after the virtual turbinectomy, and in ENS patients (0.11 Pa·s/mL) did not differ significantly from each other (*p* = 0.16–0.49). Although the virtual resection was more extensive, no statistically significant additional reduction in resistance was detected compared with the actual surgery; with six patients, this does not exclude an additional reduction. The next variable was the airflow rate (mL/s) through the inferior, middle, and superior regions of a coronal cross-section at 45% of the length of the nasal cavity. In the inferior region, the airflow rate increased after the actual surgery and increased further after the virtual turbinectomy, whereas in ENS patients it did not differ from baseline (42.0 ± 26.5 at baseline vs. 82.0 ± 27.6 after the actual surgery vs. 130.0 ± 41.7 after the virtual surgery vs. 36.6 ± 25.2 in ENS; baseline vs. actual surgery, *p* = 0.003; actual vs. virtual surgery, *p* = 0.002). Interestingly, and potentially relevant to explaining the causes of empty nose syndrome, in the middle region the airflow rate in ENS patients was higher than in all the other groups (48.3 ± 32.8 at baseline vs. 57.0 ± 29.7 after the actual surgery vs. 44.3 ± 35.1 after the virtual surgery vs. 103.8 ± 49.7 in ENS; *p* < 0.001 versus baseline and versus virtual surgery, *p* = 0.002 versus actual surgery). These results in ENS patients may be explained by the diversion of airflow away from the inferior region and toward the middle region. It remains unclear, however, why the virtual inferior turbinectomy did not produce a similarly imbalanced flow distribution, or why the absence of the inferior turbinate did not divert airflow away from the middle turbinate regions, as observed in the ENS cohort.

### 3.3. Ostiomeatal Complex (OMC)

The aim of the computational fluid dynamics (CFD) study by Wofford et al. [16] was to estimate how antrostomy size and particle size affect the topical delivery of medication to the maxillary sinus (MS) following functional endoscopic sinus surgery. The methodology involved building sinonasal reconstructions from the CT scans of four chronic rhinosinusitis (CRS) patients, both before and after FESS, supplemented by virtual models representing varying antrostomy sizes. The simulations showed that FESS increased both MS ventilation and subsequent drug delivery compared with the pre-operative state, in which drug delivery was predicted to be negligible unless an accessory ostium was present. The analysis confirmed that the largest virtual antrostomy consistently resulted in the greatest deposition of both nebulized and sprayed drugs across all subjects. The most important finding of the study was that MS drug delivery was strongly correlated with particle size. Peak MS deposition for nebulized particles occurred around the 5–18 µm range, whereas for sprayed particles, optimal deposition was observed with sizes ranging from 5 to 20 µm. These findings suggest that FESS improves topical drug delivery to the maxillary sinus and that the choice of particle size affects how much drug reaches it; the therapeutic effect was not modeled.

In the first of their studies, Chung et al. [20] focused on the effect of uncinectomy alone, considered one of the basic components of FESS. Using CFD, the authors constructed two numerical domains based on CT-derived models: one with a preserved uncinate process (Case A) and one in which the uncinate process was virtually removed (Case B). The virtual intervention was performed using CAD software. High-resolution tetrahedral–prismatic meshes were generated in ANSYS Meshing, and the simulations were carried out in ANSYS Fluent using an unsteady breathing cycle and an SST k–ω turbulence model. Flow visualizations, such as velocity maps and streamline patterns, were created during post-processing to characterize the local airflow behavior. The analysis showed that removing the uncinate process caused a specific local change in airflow by causing the formation of a pair of counter-rotating vortices in the ostiomeatal complex (OMC). Although uncinectomy produced a marked up to one-hundred-fold increase in the maximum air velocity through the maxillary ostium, overall maxillary sinus ventilation—estimated from the reduction rate of nitric oxide (NO) concentration—increased only slightly. Specifically, the rate of NO reduction rose from 0.6% (Case A, preserved uncinate process) to 1.3% (Case B, removed uncinate process) during inspiration. This led to the hypothesis that uncinectomy itself is unlikely to enhance maxillary sinus ventilation noticeably. The second study by Chung et al. [27] involved a patient who developed polypoid changes in the maxillary sinus after a more radical procedure, namely, middle meatal antrostomy (MMA). Whereas earlier research had indicated that MMA markedly increases ventilation—for example, reducing NO concentration from 100% to 38.2%, compared with the minimal reduction seen after uncinectomy alone—the simulations in the later work revealed an important finding: the epithelial surfaces where the polyps developed, located in the lower, protruding zone of the maxillary sinus, were characterized by air stagnation throughout the entire respiratory period. The velocity in the mentioned region was found to be extremely low, on the order of 4 × 10^−4^ m/s. The authors’ hypothesis is that the lack of an effective washout mechanism in these stagnant regions, together with increased exposure to particles after surgery, may lead to chronic irritation and polypoid changes. Together, these two studies by Chung et al. [20,27] (2016, 2018) illustrate how anatomical modification through FESS alters airflow mechanics and how the long-term consequences of these dynamic changes—enhanced exposure versus local stagnation—may contribute to disease pathogenesis.

The study by Saibene et al. [36] used advanced time-resolved computational fluid dynamics (DNS-based CFD) to provide a comparative theoretical basis for standard endoscopic medial maxillectomy (EMM) and its modified, tissue-sparing version (M-EMM). The simulations were based on a model of a patient with normal sinonasal anatomy, from which two virtual postoperative anatomies were created: EMM, involving removal of the entire inferior turbinate, and M-EMM, which critically spared the anterior portion of the inferior turbinate and the medial maxillary sinus wall. EMM resulted in a 72.3% increase in airflow rate on the operated side, which led to marked flow disturbance and reduction in contralateral airflow. In contrast, M-EMM minimized these negative effects, resulting in a nearly equal flow distribution. The volumetric average of the turbulent kinetic energy (K) increased 170-fold on the operated side in EMM, compared with only 35-fold in M-EMM. The authors hypothesize that this profound flow unsteadiness in EMM, which generates high, fluctuating shear stress near the mucosa, is the likely cause of postoperative crusting and impaired air conditioning. This finding provides a theoretical, model-based argument for the tissue-sparing M-EMM technique; postoperative complications were not assessed.

The study by Moniripiri et al. [41] used unsteady (time-dependent) CFD simulations, performed in ANSYS-Fluent with a low-Reynolds k-ε turbulence model, to analyze how varying sizes of middle meatal antrostomy (MMA) and different respiratory rates affect airflow and maxillary sinus ventilation. The numerical model was derived from a realistic 3D reconstruction of the upper respiratory system of an adult male. The researchers carried out three different virtual surgical interventions bilaterally on the maxillary sinus, comparing the effects of: (1) uncinectomy only, (2) uncinectomy plus an 8 mm MMA (moderate), and (3) uncinectomy plus an 18 mm MMA (extended resection). The simulations were run over a full, cyclic breathing pattern under two conditions, rest and moderate activity. The analysis showed that post-FESS ventilation increased with the size of the MMA. For the moderate activity condition, the extended surgery (uncinectomy plus 18 mm MMA) resulted in around 16% of the inhaled flow (averaged across both nasal passages) entering the maxillary sinus, compared with 11% in the uncinectomy plus 8 mm MMA case. The greater airflow entering the maxillary sinus after the extended procedure (18 mm MMA) might increase evaporation from the sinus lining and dry the mucosa; heat and moisture transport were not modeled in this study. A counterintuitive finding was that, although the total volume of air entering the MS increased with breathing activity, the proportion of air entering the maxillary sinus relative to the total inhaled volume actually decreased with an increased respiratory rate, mainly because of greater air inertia. Furthermore, the study concluded that the quasi-steady flow assumption could predict the volume of air entering the maxillary sinuses with acceptable accuracy over the respiratory cycle.

The study by Han et al. [45] used CFD to compare four virtual surgical techniques used in the treatment of maxillary sinus pathology of a 49-year-old man with normal anatomy. The procedures were middle meatal antrostomy (MMA), MMA combined with inferior meatal antrostomy (MMA+IMA), mega-antrostomy (MEGA), and endoscopic medial maxillectomy (EMM). Four unilateral virtual interventions were performed on the right maxillary sinus to generate different models. EMM was the most invasive technique, involving resection of an abundant portion of the inferior turbinate, whereas MEGA represented a more conservative, modified approach. EMM produced the most adverse changes, with 63.6% of unilateral airflow entering the right maxillary sinus, indicating hyperventilation. This excessive airflow led to the highest decrease in air-conditioning capacity, followed by a fall in nasopharyngeal humidity (RH) from 91.7% (baseline) to 88.1% in the EMM model. In addition, EMM created regions of elevated wall shear stress (WSS) and increased water-vapor flux on the posterior maxillary wall surface, forming a stagnation zone. The authors suggest that these disturbances may contribute to postoperative dryness and crusting. Overall, the MEGA model offered physiological advantages over EMM by reducing local flow disturbances within the maxillary sinus and preserving air-conditioning function.

Güldiken Sarikaya et al. [51] analyzed the effect of a sinus-lifting procedure on parameters related to maxillary sinus ventilation. Three different virtual sinus-floor elevation procedures were performed of 4, 8, and 12 mm, respectively. Changes in pressure and velocity within the sinus were then analyzed using CFD. As the sinus elevation increased, a reduction in airflow was observed, particularly in the posterior part of the modified sinus floor; however, the overall velocity increased, which may result in greater WSS and a risk of damage to the sinus mucosa. In this single model, the sinus lift changed airflow parameters in proportion to the height of the elevation, and the authors suggest that this may adversely affect the maxillary sinus mucosa.

In their study, Abbasi et al. [54] analyzed the effect of a virtual uncinectomy and middle meatal antrostomy on maxillary sinus ventilation. Using CFD and based on the preoperative CT scan of a single patient, static pressure and airflow were measured. An endoscopic maxillary sinus procedure was simulated, with removal of the uncinate process and bilateral opening of the maxillary sinus. Before surgery, ventilation of the maxillary sinuses is minimal. As expected, after the procedure, a marked increase in maxillary sinus ventilation is observed. Increased airflow through the maxillary sinus occurs during both inspiration and expiration. Interestingly, the static pressure within the maxillary sinuses did not differ markedly between the pre- and postoperative models. The computed pressures agreed reasonably well with measurements in 3D-printed replicas of the pre- and postoperative airway, but this was a single airway.

### 3.4. Septal Perforation

The study by Burgos et al. [24] analyzed the effect of a nasal septal perforation on nasal airflow parameters using CFD. Four patients were included—two healthy and two with a deviated septum and nasal obstruction—in whom an initial CFD analysis of airflow parameters was performed on the basis of previously obtained sinus CT scans. A nasal septal perforation was then created virtually, with two models used for each patient. One model assumed a perforation in the anterior part of the septum, the other in the posterior part near the choanae. For the posterior perforation models, the results showed no marked effect of this perforation location on nasal airflow and low values of airflow through the perforation between the left and right nasal cavities (QR↔L; less than 10% in all cases). For anterior perforations, a marked cross-flow between the two sides was observed only in patients with pre-existing nasal obstruction, with QR↔L values of 31.19% and 39.62%. By comparison, the QR↔L cross-flow values in patients without prior rhinological complaints were 5.89% and 19.92%. These simulations suggest that anterior perforations alter airflow more than posterior ones, particularly in noses that are already obstructed. Symptoms and crusting were not assessed, so the study does not show that posterior perforations are clinically unimportant.

Farzal et al. [30] used CFD to analyze the effect of the shape and size of a nasal septal perforation on nasal airflow parameters. Based on a single sinus CT scan, six virtual models of septal perforation differing in size and shape were created. The authors analyzed multiple parameters, including air crossover through the perforation, wall shear, heat flux, water-vapor flux, resistance, and humidification. Across all perforation types, air crossover and wall shear increased with perforation size. The highest levels of wall shear, heat flux, and water-vapor flux occurred posteriorly, while the lowest levels were found anteriorly, within the borders of the perforation itself. The elevated wall shear and heat, together with the water-vapor flow at the posterior edges of perforations, may contribute to the bleeding and crusting often observed on the posterior surface of the nasal septal perforation; this was proposed by the authors and was not assessed clinically.

The study by Na et al. [48] analyzed the effect of virtually closing a nasal septal perforation on the improvement of nasal airflow parameters. It used sinus CT scans from 14 patients with nasal septal perforation (NSP), in whom the septal defect was closed using Virtual Surgery tools; airflow parameters were then compared using CFD between the models with the perforation and after its repair. The posterior edge of the perforation showed the greatest disruption in airflow parameters, but the net crossover airflow rate, the increased wall shear stress (WSS), and the surface water-vapor flux on the posterior surface of the NSP did not increase with, or correlate with, the size of the perforation. After the virtual surgery of the NSPs, the relative humidity, air temperature, and nasal resistance levels did not improve in either the choanae or the nasopharynx. Moreover, these parameters correlated more strongly with the surface-area-to-volume ratio—a geometric parameter associated with turbinate volume—than with the location or size of the NSP. These results may explain why such a large proportion of patients with a nasal septal perforation are asymptomatic.

### 3.5. Synechiae

The study by Senanayake et al. [38] was a proof-of-concept analysis using CFD to examine the aerodynamic impact of virtual nasal adhesions (NAs) within the nasal cavity model of a single healthy subject. Five models were created, each featuring a single solid cylindrical adhesion 2.5 mm in diameter, placed at common postoperative sites such as the internal nasal valve (INV). The CFD analysis showed that the NAs did not meaningfully change the overall volume of airflow through the nasal cavity. What synechiae did produce was marked local disruption of airflow and mucosal cooling in the affected region. This was most pronounced for anteriorly located NAs, and especially those at the INV, which caused the greatest cooling disturbance and raised the pressure difference by 42% relative to the no-adhesion control at the same imposed flow. The authors noted that the areas of lost cooling may coincide with TRPM8 cold receptors and suggested that such a localized loss of cooling could be misinterpreted centrally as nasal obstruction (NAO). This was not tested: the model was based on a single healthy subject, and neither receptor activity nor symptoms were measured. The aim of the subsequent Senanayake et al. [46] study was to expand on the earlier pilot findings by repeating the analysis in four healthy adults with the same CFD methodology. The study constructed 44 computational models by placing virtual 2.5 mm cylindrical adhesions at 10 bilateral locations within each subject’s nasal cavity. The analysis confirmed that NAs caused marked disruption to airflow topology, resulting in separated, recirculating flow and stagnation zones in the wake region immediately downstream. The key finding was a statistically significant increase in mean mucosal temperature—reduced cooling—in the wake region across all adhesion models. This effect was most pronounced for the anterior inferior turbinate adhesions, which showed a mean mucosal temperature increase of 1.62 °C.

Khatri et al. [47] used CFD analysis to model the impact of synechiae (adhesions) between the middle turbinate (MT) and the lateral nasal wall, a common post-FESS complication, on sinonasal physiology. The simulations were performed on virtual models generated from the CT scan of a 25-year-old woman, using steady laminar airflow at 15 L/min. Key boundary conditions included an inspired air temperature of 20 °C entering the sinonasal cavity and an initial mucosal surface temperature set at 34 °C. The results showed that all synechiae models caused aberrant downstream airflow, leading to reduced ventilation of the ipsilateral frontal, ethmoid, and sphenoid sinuses. Notably, a 20% increase in the mass-flow-weighted average velocity was observed in the contralateral (non-adhesion) nasal cavity at coronal slice 8, an increase that correlated with enhanced ventilation of the posterior ethmoid sinus at that level. Furthermore, in the models with the most extensive adhesions (full anterior, FA, and antero-inferior, AI), the bilateral average temperature in the nasopharynx remained lower by 5% and 2.5%, respectively, compared with the no-synechiae control model. Even relatively small synechiae, such as the partial antero-inferior synechia about 2.5 mm in diameter, produced a concentrated central high-velocity airflow “jet” in the middle meatus region. In this model, synechiae, which often develop after functional endoscopic sinus surgery (FESS), markedly changed the airflow dynamics within the sinonasal region. The authors suggest that this disruption could help explain why many patients with chronic rhinosinusitis continue to experience persistent symptoms despite treatment.

### 3.6. Middle Turbinates

Dayal et al. [19] compared nasal airflow differences between inferior and middle turbinectomy. Ten patients with inferior turbinate hypertrophy were included. Using virtual surgery based on sinus CT scans, two procedures were performed in each patient: inferior turbinectomy and middle turbinectomy. Nasal airflow was then analyzed using CFD. As might be expected, in both total inferior turbinectomy (TIT) and total middle turbinectomy (TMT), nasal resistance decreased, and airflow increased. In addition, nasal heating and humidification efficiencies decreased significantly after both TIT and TMT, with the changes being more pronounced in the TIT models than in the TMT models. Of concern, in TIT, the surface area where heat flux exceeds 50 W/m^2^ decreased, whereas in TMT, it did not change significantly. The authors suggested that this may be an unfavorable effect of total inferior turbinectomy, because fewer cold receptors may be activated during airflow, and that it may contribute to the nasal obstruction reported after TIT procedures; this was not tested.

Lee et al. [18] examined the effects of varying degrees of middle turbinate resection on nasal airflow. Based on a single patient’s sinus CT model, three different extents of MT resection were performed, both unilaterally and bilaterally. The extents comprised reduction of the free edge of the MT (M1), removal of the anterior part of the MT (M2), and near-total resection (M3). With increasing excision size, nasal resistance decreased (0.9% for N-M1, 0.6% for the M1–M1 models, 1.7% for N-M2 and 2.1% for the M2–M2 model, and 5.8% for N-M3 and 10.6% for the M3–M3 model); unfavorably, however, airflow through the olfactory region decreased as the extent of resection increased, suggesting an important role of the MT in directing the airstream toward the roof of the nasal cavity and the olfactory area. The authors also noted an increase in WSS in the region of the sphenopalatine ganglion, which reached its highest values in the near-total resection (M3). They speculated that such high WSS values in this region might be related to the headaches reported after extensive resection of the middle turbinate; this was a single-case simulation, and headache was not assessed. Summarizing the overall effect of the different extents of MT resection, the authors conclude that for M1 and M2, the changes in nasal airflow parameters are small, whereas for M3, they may have an unfavorable effect. These findings may serve as an important guide for rhinosurgeons who, in some cases, reduce MT tissue during endoscopic sinus surgery.

Inthavong et al. [40] performed a virtual middle turbinate resection on the nasal model of a healthy adult (n = 1) and simulated the deposition of spherical and pollen particles at 5 and 15 L/min. At 15 L/min, deposition increased in the upper lateral region of the left cavity and, to a lesser extent, on the septum, whereas total deposition decreased at 5 L/min. The authors noted that the septal increase may have been masked by the high deposition already present before surgery. They concluded that surgical planning of middle turbinate resection should take into account a possible increase in allergen deposition on the septum, particularly in patients with aspirin-exacerbated respiratory disease.

### 3.7. Paranasal Sinuses

The study by Pourmehran et al. [53] used CFD to compare the efficiency of nebulization and nasal irrigation (bottle wash) in delivering medication to the paranasal sinuses after functional endoscopic sinus surgery (FESS). The researchers built eight virtual post-operative models based on the CT scans of a 29-year-old female patient, incorporating anatomical variables such as varying maxillary ostium sizes (from 8 mm to 25 mm) and the presence or absence of a partial middle turbinectomy (partial-MT). The numerical simulations were run in Ansys Fluent, using the Volume of Fluid (VOF) method. The researchers used specialized computational models to analyze liquid flow during nasal irrigation and to assess nebulization using high-resolution poly-hexcore meshes. The findings indicated that the surgical modifications influenced the predicted delivery outcomes. Remarkably, nebulization proved to be highly effective for the sphenoid sinuses when a partial MT procedure was performed, achieving a peak deposition efficiency of 4.02% in the left sphenoid sinus (SSL) during the VS3 scenario. In contrast, nasal irrigation proved to be the superior method for targeting the frontal sinuses, although its calculated efficiency was highly sensitive to the assumed thickness of the residual liquid layer (modeled at 50, 100, and 200 µm). These results suggest that the best delivery method may depend on the target sinus and the nature of the surgical intervention.

#### Sphenoid Sinus

The study by Bahmanzadeh et al. [15] used CFD to examine the impact of a virtual endoscopic sphenoidotomy on airflow dynamics and micro-particle deposition within the human nasal cavity and sphenoid sinus. The researchers built a realistic 3D reconstruction from the CT images of a healthy subject and then performed a virtual transethmoidal sphenoidotomy by removing the ethmoid cells and a portion of the anterior sphenoid wall. The numerical simulations were run in ANSYS-Fluent using the SIMPLE algorithm to model unsteady, laminar flow over a complete 4 s respiratory cycle. The computational fluid dynamics (CFD) analysis used a combination of mesh types to model the post-surgical anatomy. Particle deposition in the preoperative model agreed reasonably with published experimental data and simulations. By analyzing the trajectories of inhaled particles, the study found that the surgical procedure greatly enhanced airflow within the sinuses. After the surgery, the sphenoid sinus received about 16% to 18% of the total air volume inhaled. This increased airflow resulted in a marked rise in particle deposition. Specifically, around 1.5% of 10 µm particles ended up in the sphenoid sinus during normal breathing conditions. These findings suggest that 10 µm droplets may be suitable for delivering topical medications after surgery. However, the authors also pointed out that the surgery exposes the previously isolated sinus to a greater number of airborne particles and allergens.

The research conducted by Moshksayan et al. [44] investigated two techniques for accessing the sphenoid sinus: minimally invasive transnasal sphenoidotomy (TNS) and the transethmoidal technique (TES). To compare the two techniques, the authors ran advanced simulations in ANSYS Fluent, which numerically solves the Navier–Stokes equations. Flow was modeled as unsteady and laminar across a full respiratory cycle, at peak flow rates of 10, 15, and 20 L/min. Particles between 1 and 30 µm in diameter were then tracked, with drag, gravity, and Saffman lift forces all taken into account. The picture that emerged was that TNS disturbed the anatomy considerably less than TES: the airway cross-sectional area changed by at most 0.4 cm^2^ with TNS, against 1.1 cm^2^ with TES. This resulted in fewer physiological changes, and what is important in the context of allergy, the amount of particle deposition in the sphenoid sinus after the TNS procedure was much lower than after TES. For example, at a flow rate of 15 L/min, only 0.2% of 7 µm microparticles were deposited, suggesting that TNS may protect the sinuses better from bioaerosols and allergens. The authors interpret this as a lower risk of contamination of the sphenoid sinus; infection was not modeled, and particle deposition is only an indirect indicator. The overall nasal resistance remained nearly unchanged, with only a 3.4% decrease following the TNS intervention. This might favor the transnasal technique in isolated sphenoid sinus disease. However, it must not be omitted that in patients with chronic rhinosinusitis, especially with polyposis, the preferred pathway to the sphenoid sinus is through the posterior ethmoid cells.

## 4. Discussion

This review examined the use of CFD and virtual surgery methods in rhinology. Despite the diversity of the studies and the range of anatomical regions and surgical procedures analyzed, a number of common threads run through the reviewed work. The first is that virtual results were compared with an actual postoperative state in only 7 of the 41 studies, in one to seven patients each. In most of these, the virtual and the actual results agreed in the respects that were compared, and in some the simulation was used to plan the procedure. The comparisons concerned different things (postoperative anatomy, computed airflow, or symptoms), and they are discussed separately in Section 4.1. A second common thread is that in several small studies a larger resection did not improve the computed airflow parameters further. The relationship was not linear, and in some models the gain leveled off or the heat and moisture parameters worsened. The point at which this happened depended on the anatomy, the procedure, and the outcome considered, so these results do not define a general optimal extent of surgery (Section 4.3). A third point of interest is the move away from the classical approach to assessing the objective effectiveness of rhinological procedures, based on the volume of air flowing through the nose and the resistance it meets, toward parameters such as mucosal heat-and-moisture exchange, wall shear stress, and regional cooling. These factors have been proposed as better correlates of patient satisfaction and the subjective sense of nasal airflow, but this remains a hypothesis. Studies in larger patient populations with better symptom assessment, for example using dedicated scales, are still lacking.

### 4.1. Agreement Between Virtual and Actual Surgery

One of the most important questions regarding the usefulness of CFD in virtual surgery is whether such a simulation translates into the results achieved in the operating room. Seven studies addressed this directly, but they assessed different outcomes, so it is useful to separate three levels of evidence: postoperative anatomy, computed airflow, and symptoms. At the level of anatomy, Clipp et al. [21] compared virtual plans with postoperative CT in seven patients and found the virtual-to-postoperative landmark distance (mean 0.49 mm) to be substantially smaller than the pre- to postoperative change, indicating that the surgeon could reproduce the planned anatomy. Berger et al. [28] compared the calculated resection with the postoperative CT of one patient and reported good agreement in the cross-section of the nasal cavity. These studies show that a virtual plan can be carried out, but they tell us nothing about the resulting airflow or symptoms. At the level of computed airflow, Radulesco et al. [33] reported correlations between virtual and real septoplasty for heat flux, total pressure, temperature, and wall shear stress, with correlation coefficients from r = 0.92 to 0.98. These came from repeated measurements in two patients, and a correlation does not show that the values agree. In one patient, Moghaddam et al. [35] found the minimal cross-sectional area and resistance to be close between the virtual and the actual operation. Odeh et al. [52] found a different pattern in six patients: resistance after the virtual total turbinectomy did not differ significantly from that after the actual surgery, but the airflow rate through the inferior region was higher (130.0 ± 41.7 vs. 82.0 ± 27.6 mL/s, *p* = 0.002). The virtual resection was more extensive than the real one, so this difference may partly reflect the surgical extent rather than an error of the model. At the level of symptoms, SanMiguel-Rojas et al. [23] reported SNOT-22 scores before and after operations planned with CFD in two patients, Radulesco et al. [33] reported NOSE scores in two patients, and Burgos et al. [26] described resolution of symptoms in two patients without using a scale. The patients improved in all three studies, but these are descriptive data without control groups, so they cannot show whether the simulation predicted the improvement. The designs also differed: in three studies [21,23,26] the operation was carried out after the virtual plan had been made, whereas in three others [33,35,52] the virtual operation was performed retrospectively on the preoperative CT of patients who had already been operated on. None of the seven studies had a control arm, and the source articles do not describe a prospective design. Most of the 41 studies compared only physical parameters, and the patients’ subjective experience, which ought to be the ultimate endpoint for rhinological procedures, was rarely assessed. So far, the evidence shows that virtual plans can be reproduced surgically and that, in a few patients, computed airflow was similar after actual and virtual surgery. Whether CFD-based planning improves patient outcomes has not yet been shown.

The two kinds of data, computed parameters and the patient’s experience, could be linked in future protocols. In our opinion, every patient should complete a validated symptom questionnaire (for example NOSE or SNOT-22) before surgery and at a fixed time afterward, and the CFD parameters should be computed with the same protocol from the preoperative and, when available, the postoperative CT. The change in each parameter could then be related to the change in the score patient by patient, and the simulated result should be recorded before the operation, so that prediction and outcome remain separate. This would show whether the parameters discussed in Section 4.2 track symptoms at all. A recent narrative review of virtual septoplasty [12] reported that CFD metrics correlated more strongly with validated symptom scores than resistance measures did, although the reported correlations varied and depended on nasal cycle control and methodological heterogeneity.

### 4.2. Shift in Nasal Airflow Analysis

In recent years, a change has been observed in the way the physical parameters of nasal airflow and their influence on the sense of patency are assessed. The typical approach focused on treating nasal obstruction as a problem of cross-sectional area and resistance, yet it has been proposed that these bulk parameters explain symptoms only partially. Siu et al. [34] found that total inferior turbinectomy lowered wall shear stress, Dayal et al. [19] found that it reduced the mucosal surface area exposed to heat fluxes above 50 W/m^2^, and in Siu et al. [39] total heat flux was lowest after turbinectomy. The authors interpreted these changes as reduced mucosal cooling, which might blunt the thermoreceptor activation that is thought to underlie the sensation of a patent airway. This is still a hypothesis, because wall shear stress is not a measure of heat flux, heat flux was not calculated in [34], and none of these studies assessed symptoms. It has been suggested that a purely quantitative approach to nasal surgery, without due regard for the nasal mucosa and its receptors, may contribute to empty nose syndrome (ENS), in which patients perceive obstruction despite an objectively wide, low-resistance cavity. Studies of intranasal adhesions are consistent with this hypothesis but do not test it. Senanayake et al. [38,46] found in healthy models that small virtual adhesions did not change the overall airflow much but disturbed airflow and mucosal cooling locally; in the single-subject study, the pressure difference at the same imposed flow was also 42% higher with an adhesion at the internal nasal valve [38]. The authors linked the loss of cooling to TRPM8 cold receptors, but neither receptor activity nor symptoms were measured. The findings of Odeh et al. [52] also argue against a single explanation: the virtual total turbinectomy did not reproduce the shift in airflow toward the middle region that was seen in patients with ENS. If the hypothesis is correct, even a structurally successful operation could fail if it affects the regional cooling and conditioning that underlie the sense of adequate nasal airflow, but this remains to be shown.

### 4.3. More Does Not Necessarily Mean Better

In several of the reviewed studies, a greater surgical extent did not produce better computed results, and the simulations showed that less invasive procedures achieved similar airflow parameters. In two of three patients, Xiong et al. [32] found that resections 35% and 56% smaller than a total inferior turbinectomy produced similar airflow improvements, whereas leaving the narrowest segment untreated, even by 3.4–7.8% less resected volume, reduced airflow through the obstructed side by 22–32%. Burgos et al. [25] found, in a single model patient, that mucotomy alone was comparable to septoplasty for improving patency. In the context of sinus surgery, the conclusions were similar. In a single normal model, Saibene et al. [36] found that the tissue-sparing modified endoscopic medial maxillectomy caused less flow disturbance and turbulent kinetic energy than the standard operation (a 35-fold versus a 170-fold increase on the operated side). Han et al. [45] found in one model that a mega-antrostomy maintained air-conditioning capacity, which was compromised in endoscopic medial maxillectomy (nasopharyngeal humidity fell from 91.7% to 88.1%). Similarly, in Waldmann et al. [42] and Macellari et al. [50], lower resistance was obtained without complete tissue removal, in one and three patients respectively. In Odeh et al. [52], no statistically significant additional reduction in resistance was detected after the virtual total turbinectomy compared with the less extensive actual surgery, but only six patients were studied. In several simulations, more extensive resections were associated with reduced heat and moisture exchange, stagnation zones, or high wall shear stress [19,34,36,45]. The authors related these findings to crusting or ENS-like symptoms, but such outcomes were not assessed. The extent that looks best also depends on the endpoint. In the study by Rüttgers et al. [49], different weights given to airflow resistance and to air heating led to different resection plans in the same patients, from a nearly complete inferior turbinectomy to only slight reductions of both turbinates. A larger resection lowers resistance but may reduce heating and humidification, so there is no single optimal extent. Some studies gave numerical criteria, for example a resection 35–56% smaller than a total turbinectomy [32], up to 3 cm of posterior septum [31], a minimal cross-sectional area of 0.20 cm^2^ [37], a pressure drop below 1 Pa/mm [28], and heat flux above 50 W/m^2^ [19,29]. These figures are specific to the study and the model in which they were derived and should not be used as clinical thresholds. The results suggest that CFD could support decisions about the extent of surgery in the individual patient by showing what a given resection is expected to change for a chosen endpoint. The indication for surgery remains a clinical decision.

### 4.4. What the Future Holds

The most recent studies point to one possible direction in which CFD and virtual surgery technology may develop. Some of them try to automate the analysis of imaging studies and CFD simulations. Machine-learning and optimization techniques may shorten the analysis and make the method easier to use, but in the reviewed studies they were applied to one to three patients [42,49,50], and none compared the proposed plans with clinical outcomes. Waldmann et al. [42] combined computational fluid dynamics (CFD) with a machine-learning model to predict useful shapes more quickly than full numerical simulation. Rüttgers et al. [49] used a reinforcement-learning algorithm to propose a resection volume while considering different goals. Macellari et al. [50] applied adjoint optimization to create sensitivity maps for surgeons. One important finding from these research papers is that the main goals—reducing airflow resistance and maintaining air heating—sometimes conflict with each other. This means that no single measure can define the best outcome. These tools may help to balance these goals for each patient. For now, they should be regarded as research tools and planning aids; their use as decision-support systems in preoperative planning or surgical education would require validation against clinical outcomes.

### 4.5. Limitations

The limitations of this review fall into two groups: those arising from how the review itself was conducted, and those inherent in the studies it summarizes.

One limitation of the review process is the inclusion of only two databases, PubMed and Scopus, and the restriction of the search to English-language articles published from 2015 onwards. We did not search grey literature, conference proceedings or trial registries, and we did not contact authors for unpublished data. The authors of one report were contacted only to obtain its full text. Two of the included studies are conference papers [21,28]; they were found through the database search, but conference proceedings were not searched systematically, so other such reports may have been missed. Reference lists of the included studies were not screened systematically. Although the search was designed around the research questions, single relevant studies may have been missed, for example because of indexing. No review protocol was registered. The research questions, eligibility criteria and search strategy were agreed by the authors before the search, but informally, and no written protocol was published, so we cannot document that they were fixed in advance. In keeping with scoping review methodology, we did not formally appraise the methodological quality or the risk of bias of the included studies, so the findings reported here are mapped rather than weighted by study quality. Data were charted by two authors, and although the entries in Table 2 and the numbers quoted in the Results were checked against the full texts, charting errors cannot be excluded. The funding of the included studies was not charted.

#### Strengths and Limitations of the Available Evidence

Another issue is the small sample sizes and the lack of control groups and control values in the included studies. Most of the reviewed work was based on computed tomography from a small number of patients, and in several studies on a single scan. Because sinonasal anatomy varies widely between individuals, baseline airflow derived from one or two subjects is a weak reference against which to judge the effect of a virtual intervention. Control data were also handled inconsistently: some authors compared postoperative models with the same patient’s preoperative state, others with a separate healthy cohort, and others with published normative values. Where healthy controls were used, cohort size ranged from two subjects to several dozen. Several publications also share the same subjects (Section 3.1), so the number of studies overstates the number of independent samples.

CFD methodology varied across included studies at almost every step of the workflow. Segmentation was performed manually in some studies and semi-automatically in others, with different thresholding conventions and different software; mesh type, mesh density, and mesh-independence testing were reported inconsistently and sometimes not at all. Simulations were variously run as steady or unsteady, laminar or turbulent, and where turbulence was modeled, the choice of model differed between studies. Inlet conditions also differed, affecting flow parameters, so absolute values reported by different groups cannot be compared directly. Table 2 documents these differences, but we did not attempt to standardize them or to judge which choice was appropriate, which is outside the scope of a scoping review. Values from different studies should therefore not be compared directly; comparisons within one study, for example before and after a virtual intervention in the same model, are more meaningful.

Modeling assumptions were common to nearly all included studies. The nasal walls were treated as rigid, and none of the 41 studies used fluid–structure interaction, so the movement of soft tissue, such as dynamic collapse of the nasal valve on inspiration, was not represented. Some studies represented the mucus and submucosal layers in simplified form, as a thermal resistance in the wall model (Section 3.1), but none modeled mucus transport or mucociliary function, and the nasal cycle and the vascular response of the turbinate mucosa were not modeled. Mucosal swelling, inflammation, and secretions enter the models only as they appear on a single CT scan, and the reconstructed geometry also depends on the CT technique and on the position of the patient during the scan. Simulations therefore describe a single frozen anatomical state, in one phase of the nasal cycle, rather than a physiologically fluctuating airway. This limits how far the results can be applied to a patient whose symptoms vary over time. Only 7 of the 41 studies compared their simulations with an actual postoperative state (in one to seven patients each) and 6 with experimental measurements or published simulations (Section 3.1), so the accuracy of the computed values relative to the real airway is largely unverified.

Absence of patient-reported endpoints. Most studies compared physical parameters before and after virtual intervention without any measure of how the patient felt. Validated patient-reported measures were used in only three studies: SNOT-22 [23] and NOSE [33], in two patients each, and NOSE with a visual analog scale in six patients [52], where the scores describe the actual operation rather than the virtual one. One further study described resolution of symptoms without a scale [26]. Where symptom scales were used, they were applied in small numbers of patients, at short follow-up, and without blinding. We identified no prospective study in which a CFD-derived plan altered the surgical decision and the outcome was then assessed against a control arm. Whether virtual surgery improves patient-reported outcomes therefore remains untested.

Absence of validated thresholds and unit conventions. Several of the parameters used across these studies to define a favorable result—the mucosal surface area exposed to heat flux above 50 W/m^2^, or a minimal cross-sectional area of 0.20 cm^2^—were derived from single centers and have not been externally validated. They come from small samples or single models and should not be read as clinical thresholds. Nasal resistance was reported in incompatible units between studies. For these reasons, no quantitative synthesis of the results was possible, and the review is necessarily narrative.

Scope restrictions. Pediatric (<18 years), sleep apnea, orthognathic, environmental and toxicological studies were excluded, as were studies of the lower respiratory tract. These restrictions kept the review focused on adult rhinological surgery, but they exclude a body of methodologically relevant CFD work, and two included studies sit at the edge of this boundary: one on pollen deposition after middle turbinectomy [40] and one on sinus floor elevation, for which the indication is dental [51].

Despite the rapid development of CFD over the past decade, the method is still not a tool used in the daily practice of otolaryngologists and head and neck surgeons. The barrier lies in the complexity of processing computed tomography scans and the time required to analyze the anatomical reconstructions—time that rhinosurgeons rarely have in routine clinical practice. A potential solution would be fully automated, rapid CT-analysis pipelines that return ready-to-use results indicating the anatomical sites that may require surgical correction.

## 5. Conclusions

Computational fluid dynamics, combined with software that enables the design of surgical procedures, is a promising way to simulate the effects of rhinological procedures on airflow. The multitude of procedures that can be performed, together with the variability of the anatomy within the nasal cavity and paranasal sinuses, compels the surgeon to adopt a precise and individualized approach to rhinological surgery. As technology develops and the workload required to simulate procedures decreases, virtual surgery tools may provide useful support for planning, which might benefit patients; this has not yet been shown. In several small simulation studies included in this review, a larger resection did not improve the computed airflow parameters further, and in some it worsened heat and moisture exchange and regional cooling. The extent that appeared best depended on the anatomy, the procedure, and the outcome chosen, so the results do not define a general optimal extent of resection, and whether excessive resection leads to empty nose syndrome (ENS) was not tested. Besides the quantitative improvement of nasal airflow, parameters such as mucosal heat-and-moisture exchange, wall shear stress, and regional cooling deserve attention as possible correlates of the patient’s subjective sense of a patent nose. This hypothesis needs to be tested in patients who complete validated questionnaires such as NOSE or SNOT-22, and the approach might then help patients who, despite a successful surgery from the surgeon’s perspective, still experience problems with nasal patency. Only a few studies compared virtual results with actual postoperative anatomy, airflow, or symptoms, in one to seven patients each; larger prospective studies with control groups are still lacking. Another important issue requiring standardization is the methods of analyzing imaging studies with CFD, which differ between papers and may considerably affect the results obtained. Nevertheless, virtual surgery methods using CFD may become a valuable addition to the surgeon’s daily practice. At present they are a research and planning aid; the decision rests with the surgeon, and their effect on surgical outcomes remains to be shown.

## Figures and Tables

**Figure 1:**
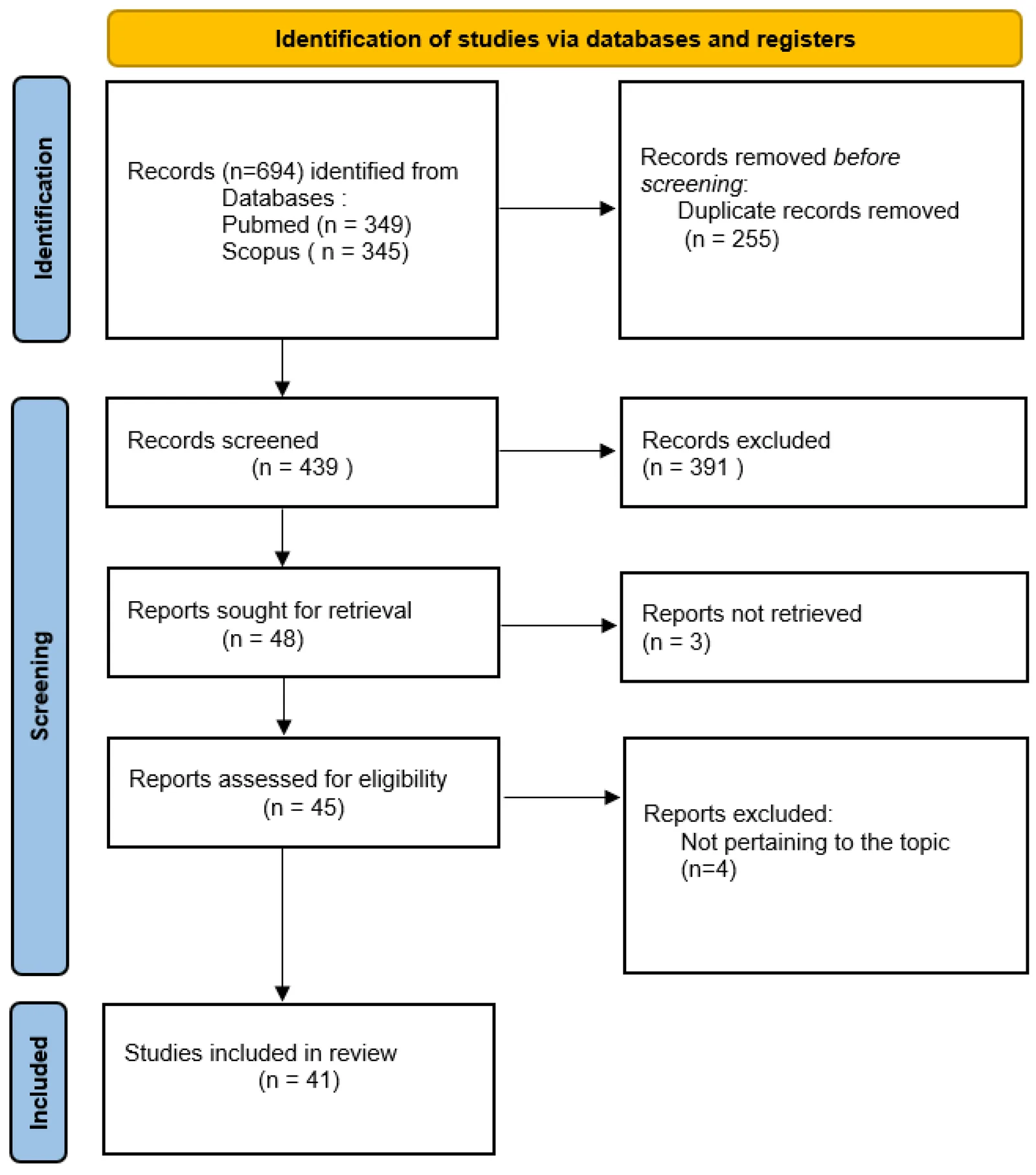
PRISMA 2020 flow diagram of the study selection process.

**Table 1:** Summary of studies applying computational fluid dynamics (CFD) and virtual surgery in rhinosurgery.

Study	Patients/Models	Anatomical Region	Virtual Intervention	Outcomes
Hariri et al., 2015 [14]	5	Inferior turbinate	Inferior turbinoplasty/turbinate resection middle vs. bottom	Nasal resistance fell after all virtual reductions, with no marked difference between bottom and medial resection; the largest fall occurred in patients with the highest presurgical resistance in the turbinate region.
Bahmanzadeh et al., 2015 [15]	1	Sphenoid sinus	Sphenoidotomy	Sphenoidotomy increased airflow into the sphenoid sinus and, with it, micro-particle deposition there.
Wofford et al., 2015 [16]	4	Maxillary sinus	Different antrostomy sizes	Drug delivery to the MS was highest with the largest antrostomy and depended on particle size.
Simmen et al., 2015 [17]	1	Inferior turbinate and nasal valve area	Pyriform turbinoplasty (with lateral nasal wall lateralization)	Peak velocity (up to 2.5 m/s) in the nasal valve area; after the virtual procedure the septum–inferior turbinate distance increased by about 2 mm, and after the extended procedure meatal airspeed fell from 0.3 to 0.1 m/s, with little change in the posterior airflow.
Lee et al., 2016 [18]	1	Middle turbinate	Middle partial turbinectomy—3 types	Removing the anterior inferior part of the MT and keeping its posterior part changed airflow little; the authors consider this useful for planning partial MT resection.
Dayal et al., 2016 [19]	10	Inferior and middle turbinate	Total inferior turbinectomy and total middle turbinectomy	Both TIT and TMT lowered resistance and increased airflow. The mucosal area with heat flux above 50 W/m^2^ fell after TIT and did not change significantly after TMT, which the authors linked to weaker stimulation of cold receptors. Heating and humidification efficiency fell significantly after both procedures, more after TIT.
Chung et al., 2016 [20]	1	Uncinate process	Uncinectomy	Maxillary sinus ventilation was similar on inspiration and expiration, with or without the uncinate process; uncinectomy alone did not increase it appreciably.
Clipp et al., 2018 [21]	7	Septum/Inferior turbinates	Septoplasty/Inferior turbinoplasty	An experienced clinician tested the tool for usability and accuracy; the results were rated satisfactory, and the virtual plans closely matched the postoperative CT scans.
Lee et al., 2018 [22]	5	Inferior turbinate	Inferior turbinoplasty—6 different types	ITR along the whole turbinate gave the best improvement in airflow and resistance in all 5 patients. The best site for partial ITR varied between patients and even between the two sides of one patient, and in some patients posterior ITR was more effective than anterior ITR.
Sanmiguel-Rojas et al., 2018 [23]	2	Septum/Inferior turbinates	Septoplasty/Inferior turbinoplasty	Both patients were operated on according to the CFD-guided virtual plan and reported high satisfaction.
Burgos et al., 2018a [24]	4	Septum	Creation of septum perforation	A created NSP markedly altered airflow only in noses with preexisting obstruction, not in normal noses; the authors relate this to the variable symptoms and the many asymptomatic patients with perforation.
Burgos et al., 2018b [25]	1	Septal deviation	Comparison of septoplasty, turbinectomy, spreader-grafts, J-flap and combination among them	Combining all procedures did not give the largest improvement; septoplasty with turbinoplasty did, and turbinoplasty alone was almost as effective as septoplasty in this simulation.
Burgos et al., 2018c [26]	2	Septum + Inferior turbinate/Ethmoid and maxillary sinus + Inferior turbinate	Septoplasty + inferior turbinoplasty/ESS + inferior turbinoplasty	Symptoms resolved after real surgery performed according to the virtual plan (qualitative)
Chung et al., 2018 [27]	1	Maxillary sinus	Polypectomy	Air velocity in the MS was much lower than in the middle meatus, and wall shear and pressure changes were negligible on the sinus mucosa where polypoid changes had developed.
Berger et al., 2019 [28]	4	Septal deviation	Assessment of surgically relevant points	An optimizer reshaped the nasal cavity at SRPs. Postoperative CT showed a larger cross-section at all SRPs, and the optimized and postoperative cross-sections differed by less than 15%.
Frank-Ito et al., 2019 [29]	2	Septum/Inferior turbinates	Septoplasty/Inferior turbinoplasty	Septoplasty alone, and septoplasty with inferior turbinectomy on the less obstructed side, reached the healthy reference ranges.
Farzal et al., 2019 [30]	1	Septum	Creation of septum perforation	Crossover flow and wall shear rose with perforation size; high wall shear, heat flux, and water-vapor flux at the posterior edge may explain the crusting usually seen there.
Li et al., 2020 [31]	1	Septum	Posterior septectomy—3 different types	Resection of up to 3 cm of the posterior septum hardly changed the airflow pattern or nasal warming, suggesting little effect on nasal function.
Xiong et al., 2020 [32]	3	Inferior turbinate	Total inferior turbinectomy vs. partial inferior turbinate reduction	Removing more tissue did not always increase the flow rate further, including on the MOS.
Radulesco et al., 2020 [33]	2	Septum	Septoplasty	No differences were found for any CFD measurement between VS and PS (*n* = 2)
Siu et al., 2020 [34]	8	Inferior turbinate	Total inferior turbinectomy vs. turbinoplasty	Turbinectomy lowered resistance slightly more than turbinoplasty, but after turbinoplasty the ventilation, pressure, and wall shear stress distributions were nearer to those of the healthy models.
Moghaddam et al., 2020 [35]	1	Septum	Septoplasty	Minimal cross-sectional area and nasal resistance predicted by the virtual operation agreed well with those after the actual operation.
Saibene et al., 2020 [36]	1	Maxillary sinus	Endoscopic medial maxillectomy—2 variants	EMM markedly disrupted nasal airflow, whereas modified EMM (M-EMM) changed it less and interfered less with air conditioning.
Campbell et al., 2021 [37]	10	Deviated septum	Creating septum deviation	A small, unrecognized CSAPV may explain persistent obstruction after septoplasty; with a narrow CSAPV, inferior turbinate reduction on the side of the deviation may increase the airflow gain of septoplasty.
Senanayake et al., 2021 [38]	1	Nasal adhesions	Creation of nasal adhesions	Adhesions hardly changed bulk airflow but markedly changed local airflow and mucosal cooling around and just behind them, most of all at the internal valve and the anterior inferior turbinate.
Siu et al., 2021 [39]	8	Inferior turbinate	Total inferior turbinectomy vs. turbinoplasty	Air conditioning changed significantly after inferior turbinate reduction, most after turbinectomy with CDA, when streams of much colder air reached the nasopharynx.
Inthavong et al., 2021 [40]	1	Middle turbinate	Middle partial turbinectomy	Resection widened the upper part of the main nasal passage; the authors suggest that planning should take into account a possible increase in allergen deposition on the septum.
Moniripiri et al., 2021 [41]	1	Ostiomeatal complex and maxillary sinus	Uncinectomy vs. 2 different antrostomies	After sinus surgery, much more air entered the MS, and the increase grew with MMA size.
Waldmann et al., 2022 [42]	1	Deviated septum	Septoplasty	Septoplasty barely changed the heating capacity of the nose, lowered total resistance by 25.3%, and redistributed the mass flux.
Tao et al., 2022 [43]	1	Deviated septum	Septoplasty	Two surgical plans (A/B): correcting vestibular obstruction and caudal septal deviation, in addition to deeper septal deviation, raised the share of inspiratory flow on one side by up to 17%.
Moshksayan et al., 2022 [44]	1	Sphenoid sinus	Transnasal sphenoidotomy vs. transethmoidal sphenoidotomy	TNS disturbed the anatomy considerably less than TES: the airway cross-sectional area changed by at most 0.4 cm^2^ with TNS, against 1.1 cm^2^ with TES. This resulted in fewer physiological changes, and what is important in the context of allergy, the amount of particle deposition in the sphenoid sinus after the TNS procedure was much lower than after TES
Han et al., 2023 [45]	1 patient (5 models)	Maxillary sinus	Different antrostomy sizes	MMA and MMA+IMA behaved like the baseline model, whereas EMM and MEGA changed sinus physiology: airflow into the MS was largest after EMM, then MEGA, and wall shear stress and water-vapor flux rose near the enlarged ostium. The authors favor MEGA and modified EMM, which allow a sufficient resection with fewer changes than EMM.
Senanayake et al., 2023 [46]	4 (44 models)	Nasal adhesions	Creating nasal adhesions	The key finding was a statistically significant increase in mean mucosal temperature—reduced cooling—in the wake region across all adhesion models. This effect was most pronounced in the anterior inferior turbinate adhesions, which showed a mean increase in mucosal temperature of 1.62 °C.
Khatri et al., 2023 [47]	1	Nasal adhesions	Creating nasal adhesions	Post-FESS synechiae between the MT and the lateral nasal wall markedly disturbed sinus ventilation downstream: the frontal, ethmoid, and sphenoid sinuses on the same side were less ventilated, and a central jet formed in the middle meatus. The effect increased with synechia size; bulk airflow was hardly affected.
Na et al., 2024 [48]	14	Septal perforation	Repair of septal perforation	Virtual NSP closure did not significantly improve RH, AT, or resistance in the choanae and nasopharynx. RH and AT depended more on SAVR, which reflects turbinate volume, than on the size and location of the perforation.
Rüttgers et al., 2024 [49]	2	Deviated septum and inferior turbinate hypertrophy	Septoplasty and turbinoplasty	Criteria: pressure loss (i) and air heating (ii). In a patient with an anterior septal deviation and a bony spur, the method targeted both sites; with equal weights it proposed a slightly smaller septal correction than the surgeon and kept the spur, which was removed when (i) was weighted more, while more weight on (ii) moved the result toward the narrower preoperative anatomy. In a second patient with enlarged turbinates, equal weights gave nearly complete inferior and moderate middle turbinate reduction; more weight on (i) added middle turbinate reduction, and more weight on (ii) gave only slight reductions of both turbinates.
Macellari et al., 2024 [50]	3	Deviated septum	Septoplasty	Surface sensitivity maps identified all functionally relevant anatomical changes automatically; ENT surgeons reviewed the suggested interventions, and the method was robust to several computational settings.
Güldiken Sarıkaya et al., 2025 [51]	1 patient (3 models)	Maxillary sinus	Sinus lift	Sinus lifting for dental implants may impair sinus drainage, especially with greater elevation, and faster airflow in the smaller sinus may increase the risk of mucosal damage.
Odeh et al., 2025 [52]	6	Inferior turbinate	Total inferior turbinectomy	The virtual turbinectomy did not reproduce the distorted airflow seen in ENS, and resistance did not differ significantly from that after the actual surgery; the ENS cohort had a wider inferior airway CSA than the operated patients. The authors regard ENS as multifactorial rather than caused by aggressive turbinate reduction alone. After the actual surgery, the NOSE score fell from 72.5 ± 13.2 to 10.8 ± 9.8 and the unilateral visual analog scale score from 6 ± 2.56 to 1.2 ± 1 (both *p* < 0.001).
Pourmehran et al., 2025 [53]	1 (8 models)	Paranasal sinuses	FESS	Nebulization deposited more drug than BW in most sinuses, especially after partial MT resection, and was more efficient in the maxillary and sphenoid sinuses; BW was better for the frontal sinuses. Treatment outcomes were not assessed.
Abbasi et al., 2025 [54]	1 (+1 model)	Maxillary sinus	Uncinectomy and antrostomy	Uncinectomy and MMA markedly improved maxillary sinus ventilation, more so at higher inspiratory flow; pressure in the sinus stayed nearly uniform except near the ostium.

Abbreviations: AT, air temperature; BW, bottle wash; CDA, cold, dry air; CFD, computational fluid dynamics; CSA, cross-sectional area; CSAPV, post-valve cross-sectional area; EMM, endoscopic medial maxillectomy; ENS, empty nose syndrome; ENT, ear–nose–throat; ESS, endoscopic sinus surgery; FESS, functional endoscopic sinus surgery; IMA, inferior meatal antrostomy; ITR, inferior turbinate reduction; MEGA, mega-antrostomy; MMA, middle meatal antrostomy; MOS, most obstructed side; MS, maxillary sinus; MT, middle turbinate; NSP, nasal septal perforation; PS, postoperative state; RH, relative humidity; SAVR, surface area-to-volume ratio; SRP, surgically relevant point; TES, transethmoidal sphenoidotomy; TIT, total inferior turbinectomy; TMT, total middle turbinectomy; TNS, transnasal sphenoidotomy; VS, virtual surgery. Patients/models: number of patients or healthy subjects whose imaging was modeled; the number of virtual models is given in parentheses when it differs, and comparator groups (for example, healthy controls) are not counted. Terminology: turbinectomy, removal of turbinate tissue (total or partial); turbinoplasty, volume reduction that preserves part of the turbinate; reduction, either procedure.

**Table 2:** Methodological characteristics of the included studies.

Study	Geometry (n)	CFD Software/Solver	Flow Regime	Turbulence	Heat/Humidity	Validation
Hariri 2015 [14]	CT (5 pt)	Fluent 14.0; Mimics 16.0 (mesher not named)	Steady (15 L/min)	Laminar	Yes (heat + humidity; inlet 20 °C, 50% RH; wall 32.6 °C, 100% RH)	None
Bahmanzadeh 2015 [15]	CT (1)	Fluent 14.0; ICEM-CFD (SIMPLE)	Unsteady (4 s cycle)	Laminar	No (particle)	vs experiment and prior CFD (particle deposition)
Wofford 2015 [16]	CT (4 pt)	Fluent 12.1; Mimics; ICEM-CFD	Steady	Laminar	No (drug deposition)	None
Simmen 2015 [17]	CT (1 pt)	Fluent (ANSYS Workbench 14); ITK-SNAP 3.0.0	Unsteady (4.2 s breathing cycle; in vivo pressure and volume)	Not stated	Yes (heat + humidity; inspiration 20 °C, 30% RH; expiration 37 °C, 100% RH; no thermal results reported)	None
Lee 2016 [18]	CT-derived (1, symmetric)	Fluent 16; Mimics + Rapidform; ICEM	Steady (+ unsteady)	Laminar	Yes (heat; inlet 20 °C; wall 34 °C)	None
Dayal 2016 [19]	CT (10 pt)	Fluent 14.0; Mimics 16.0 (mesher not named)	Steady (15 L/min)	Laminar	Yes (heat + humidity; inlet 20 °C, 50% RH; wall 32.6 °C, 100% RH)	None
Chung 2016 [20]	CT (1)	ANSYS-Fluent 14.5; VWORKS	Unsteady (cyclic)	SST k–ω (low-Re)	No (NO washout)	None
Clipp 2018 [21]	CT (7 models; cohort 10 pt)	Lattice-Boltzmann (Autodesk, GPU); 3D Slicer	Steady (Δp BC)	Not stated (LBM)	No	gLBM vs. Fluent (1 case); virtual vs. real post-op septal geometry (n = 7)
Lee 2018 [22]	CT (5 pt)	Fluent; Amira; ICEM	Steady (Δp = 15 Pa)	Laminar	No	None
Sanmiguel-Rojas 2018 [23]	CT (2 pt)	MeComLand + DigBody	Steady (Δp BC)	Laminar	No	Actual surgery: SNOT-22 (n = 2), descriptive
Burgos 2018a [24]	CT (4 pt)	MeComLand + DigBody (ITK)	Steady	Laminar	Yes (heat; inlet 20 °C; wall 37 °C)	None
Burgos 2018b [25]	CT (1)	MeComLand + DigBody (OpenFOAM; ITK)	Steady	Laminar	No	None
Burgos 2018c [26]	CT (2 pt)	MeComLand + DigBody (ITK)	Steady	Laminar	No	Actual surgery after virtual plan: symptom resolution (n = 2), qualitative
Chung 2018 [27]	CT (1)	ANSYS-Fluent 18.2; VWORKS	Unsteady (cyclic)	SST k–ω (low-Re)	No	Qualitative comparison with published PIV data
Berger 2019 [28]	CT (4 pt: 1 healthy + 3 NAO)	Sailfish CFD (GPU-LBM); Python; ParaView	Steady (velocity BC)	LES (Smagorinsky)	No	LBM validated vs. LDA in earlier work; post-op CT (n = 1)
Frank-Ito 2019 [29]	CT (2 pt; vs. 47 healthy)	Fluent 16.1; Mimics; ICEM-CFD	Steady	Laminar	Yes (heat; inlet 20 °C; wall 32.6 °C)	vs healthy controls (n = 47)
Farzal 2019 [30]	CT (1)	Fluent 15.0; Mimics; ICEM; Fieldview	Steady	Laminar	Yes (heat + humidity; inlet 20 °C, 50% RH; 32.6 °C, 100% RH)	None
Li 2020 [31]	CT (1)	Fluent 14.0; Mimics + Geomagic; ICEM	Quasi-steady	RNG k–ε	Yes (heat; inlet 20 °C; wall 34 °C)	None
Xiong 2020 [32]	MRI (3 pt)	Fluent; NX + MATLAB; ICEM-CFD	Steady (Δp = 30 Pa)	Laminar	Yes (heat + humidity; inlet 20 °C; mucosa 32.6 °C)	None
Radulesco 2020 [33]	CT (2 pt)	Star-CCM+; ITK-Snap + ImageJ	Unsteady (cyclic)	Laminar	Yes (heat; inlet 19 °C; wall 34 °C)	Virtual vs. actual post-op CT model (r = 0.92–0.98, n = 2); NOSE descriptive
Siu 2020 [34]	CT (8 pt) + 2 healthy	ANSYS-Fluent 2019R1; 3D Slicer; Geomagic Wrap	Steady (15 L/min)	Laminar	No	vs healthy controls (n = 2)
Moghaddam 2020 [35]	CT (1 pt + 47 healthy)	ANSYS Fluent 18.1; Mimics; ICEM; MATLAB	Steady	Laminar	No	Actual post-op state (n = 1); vs. 47 healthy
Saibene 2020 [36]	CT (1)	OpenFOAM; 3D Slicer	Unsteady (DNS, 0.6 s)	None (DNS)	No	None
Campbell 2021 [37]	CBCT (10 healthy; 50 models)	Fluent 14.0; Mimics + Sculptor Morph; ICEM	Steady (Δp = 15 Pa)	Laminar	No	None
Senanayake 2021 [38]	CT (1)	ANSYS Fluent 20R1; 3D Slicer; SpaceClaim	Steady (15 L/min)	Laminar	Yes (heat + humidity; inlet 20 °C, 35% RH; submucus wall model)	None
Siu 2021 [39]	CT (8 pt) + 2 healthy	ANSYS-Fluent 19.2; 3D Slicer; Geomagic Wrap	Steady (15 L/min)	Laminar	Yes (heat + humidity; AA 25 °C/35% RH, CDA 12 °C/13%, HHA 40 °C/80%; wall 34 °C)	vs healthy controls (n = 2)
Inthavong 2021 [40]	CT (1)	ANSYS-Fluent 2020R1; 3D Slicer	Steady (5 & 15 L/min)	Laminar	No (particle)	None
Moniripiri 2021 [41]	CT (1)	ANSYS-Fluent; Mimics; ICEM-CFD	Unsteady (4 s cycle)	Low-Re k–ε (RANS)	No	vs previous CFD study
Waldmann 2022 [42]	CT (1)	m-AIA (Thermal LBM + Level-Set); 3D Slicer (ML)	Steady (volume-flux BC)	Not stated (thermal LBM)	Yes (heat; inlet 20 °C; wall 36 °C)	Pipe test vs. published DNS; nasal case not validated
Tao 2022 [43]	CT (1)	Solver not named (DPM/EIM via UDF)	Steady (15 & 60 L/min)	Laminar + Transition SST	No (particle)	DPM validated in earlier work; trends vs. published in vitro data
Moshksayan 2022 [44]	CT (1)	ANSYS-Fluent; ICEM-CFD	Unsteady (4 s cycle)	Laminar	No (particle)	vs published experiments and simulations (particle deposition)
Han 2023 [45]	CT (1; 5 models)	ANSYS/Fluent 2021R2; Mimics + Geomagic; CardinalSim	Steady (250 mL/s)	Laminar	Yes (heat + humidity; inlet 25 °C, 35% RH; wall model)	None
Senanayake 2023 [46]	CT (4 healthy; 44 models)	ANSYS Fluent 20R1; 3D Slicer; Geomagic Studio	Steady (15 L/min)	Laminar	Yes (heat + humidity; inlet 20 °C, 30% RH; submucus wall model)	None
Khatri 2023 [47]	CT (1)	ANSYS Fluent 19.2; 3D Slicer; Geomagic Studio	Steady (15 L/min)	Laminar	Yes (heat + humidity; inlet 20 °C, 35% RH; submucus wall model)	None
Na 2024 [48]	CT (14 pt)	ANSYS/Fluent R21.2; Fluent Meshing	Steady (250 mL/s)	Laminar	Yes (heat + humidity; inlet 25 °C, 35% RH; wall model)	Method validated in authors’ earlier studies
Rüttgers 2024 [49]	CT (2 pt)	m-AIA (Thermal LBM + Level-Set, GPU); ML seg.	Steady (250 mL/s)	Not stated (thermal LBM)	Yes (heat; inlet 20 °C; wall 36 °C)	None
Macellari 2024 [50]	CT (3 pt)	OpenFOAM (adjoint); 3D Slicer	Steady (15 L/min)	SST k–ω (RANS)	No	No independent validation; feasibility reviewed by ENT surgeons
Güldiken Sarıkaya 2025 [51]	CBCT (1)	Autodesk CFD 2021; i-CAT + RealGuide; Rhinoceros	Pressure-driven (Δp = 150 Pa); steady/unsteady not stated	SST k–ω	No	None
Odeh 2025 [52]	CT (6 pt) + 27 ENS	ANSYS Fluent 19.2; Amira; ICEM-CFD	Steady (Δp = 15 Pa)	Laminar	No	CFD of real post-op CT vs. virtual model (n = 6); ENS cohort (n = 27) from earlier publications
Pourmehran 2025 [53]	CT (1; 8 models)	ANSYS Fluent; Fluent Meshing 2022R1; 3D Slicer; SpaceClaim; poly-hexcore	Unsteady (nebulizer 2 L/min; irrigation jet 60 mL/s)	Laminar (DPM); SST k–ω (VOF)	No (drug/irrigation)	None
Abbasi 2025 [54]	CT (1) + 3D-printed model	ANSYS Fluent; ICEM CFD (segmentation software not named)	Steady (constant rates 3.8–42.6 L/min)	Laminar (<~20 L/min); low-Re k–ω (>~20 L/min)	No	In vitro: pressure in 3D-printed replicas (single airway)

Abbreviations: AA, ambient air; CBCT, cone-beam computed tomography; CDA, cold dry air; CFD, computational fluid dynamics; DNS, direct numerical simulation; DPM, discrete phase model; ENS, empty nose syndrome; ENT, ear–nose–throat; GPU, graphics processing unit; HHA, hot humid air; ICEM, ICEM-CFD; ITK, Insight Segmentation and Registration Toolkit; LBM, lattice-Boltzmann method; LDA, laser Doppler anemometry; LES, large-eddy simulation; ML, machine learning; MRI, magnetic resonance imaging; NAO, nasal airway obstruction; NO, nitric oxide; NOSE, Nasal Obstruction Symptom Evaluation; PIV, particle image velocimetry; pt, patients; RANS, Reynolds-averaged Navier–Stokes; RNG, renormalization group; SNOT-22, Sino-Nasal Outcome Test-22; SST, shear-stress transport; VOF, volume of fluid. “Geometry (n)” gives the imaging modality and the number of patients (pt) or healthy subjects whose imaging was modeled, with the number of virtual models where it differs; comparator groups are listed separately; reconstructions were computed-tomography–based unless stated otherwise. “Flow regime” distinguishes steady from unsteady (full breathing-cycle) simulations. “Validation” denotes comparison of the simulated results with an external reference: an actual postoperative state (postoperative imaging, CFD of postoperative CT, or symptoms after the real operation), experimental measurements, published simulations, or a healthy reference group. Comparison with a healthy group describes normal values and does not by itself validate the prediction of a surgical result. “None” means that no external comparison was reported; mesh-independence testing alone is not counted. “Heat/humidity” entries give the inlet (inspired air) and wall or mucosal conditions where reported. Other cells marked “NR” were not specified in the corresponding source article.

**Table 3:** Included studies by anatomical region and type of external comparison.

Anatomical Region (Results Section)	Studies, n [References]	Actual Postoperative State	Experiment or Published Data	Healthy Reference Only	None
Septum, alone or with turbinates (3.2)	14 [21,23,25,26,28,29,31,33,35,37,42,43,49,50]	6	1	1	6
Inferior turbinates (3.2)	7 [14,17,22,32,34,39,52]	1	0	2	4
Maxillary sinus and ostiomeatal complex (3.3)	8 [16,20,27,36,41,45,51,54]	0	3	0	5
Septal perforation (3.4)	3 [24,30,48]	0	0	0	3
Synechiae (3.5)	3 [38,46,47]	0	0	0	3
Middle turbinate (3.6)	3 [18,19,40]	0	0	0	3
Sphenoid and other sinuses (3.7)	3 [15,44,53]	0	2	0	1
Total	41	7	6	3	25

Each study is counted once, under the strongest comparison it reported (actual postoperative state, then experiment or published data, then healthy reference only). “None” includes studies whose only check was a benchmark on a simple geometry [42], validation of the method in the authors’ earlier work [48], or review by ENT surgeons [50].

## Data Availability

Data are contained within the article.

## Supplementary Materials

The following supporting information can be downloaded at: https://www.mdpi.com/article/10.3390/jcm15208069/s1, The following supporting information is provided as separate files: Table S1: Reports excluded after full-text assessment; Table S2. Search strategies as run; Table S3: PRISMA-ScR checklist.

## Abbreviations

The following abbreviations are used in this manuscript:

**AA**	ambient air
**AT**	air temperature
**BW**	bottle wash (nasal irrigation)
**CBCT**	cone-beam computed tomography
**CDA**	cold, dry air
**CFD**	computational fluid dynamics
**CRS**	chronic rhinosinusitis
**CSA**	cross-sectional area
**CSAPV**	post-valve cross-sectional area
**CT**	computed tomography
**DNS**	direct numerical simulation
**DPM**	discrete phase model
**EMM**	endoscopic medial maxillectomy
**ENS**	empty nose syndrome
**ENT**	ear–nose–throat
**ESS**	endoscopic sinus surgery
**FESS**	functional endoscopic sinus surgery
**GPU**	graphics processing unit
**HHA**	hot, humid air
**IMA**	inferior meatal antrostomy
**ITR**	inferior turbinate reduction
**LBM**	lattice-Boltzmann method
**LES**	large-eddy simulation
**LOS**	less obstructed side
**mCSA**	minimal cross-sectional area
**M-EMM**	modified endoscopic medial maxillectomy
**MEGA**	mega-antrostomy
**MMA**	middle meatal antrostomy
**MOS**	most obstructed side
**MRI**	magnetic resonance imaging
**MS**	maxillary sinus
**MT**	middle turbinate
**NAO**	nasal airway obstruction
**NO**	nitric oxide
**NOSE**	Nasal Obstruction Symptom Evaluation
**NSP**	nasal septal perforation
**OMC**	ostiomeatal complex
**PROM**	patient-reported outcome measure
**RANS**	Reynolds-averaged Navier–Stokes
**RH**	relative humidity
**RNG**	renormalization group
**SAVR**	surface area-to-volume ratio
**SNOT-22**	22-item Sino-Nasal Outcome Test
**SRP**	surgically relevant point
**SST**	shear-stress transport
**TES**	transethmoidal sphenoidotomy
**TIT**	total inferior turbinectomy
**TMT**	total middle turbinectomy
**TNS**	transnasal sphenoidotomy
**VOF**	volume of fluid
**WSS**	wall shear stress

## References

[1] Tretiakow D., Tesch K., Markiet K., Przewoźny T., Kusiak A., Cichońska D., Skorek A. (2023). Numerical analysis of the ostiomeatal complex aeration using the CFD method. Sci. Rep..

[2] Tretiakow D., Tesch K., Markiet K., Skorek A. (2022). Maxillary sinus aeration analysis using computational fluid dynamics. Sci. Rep..

[3] van Egmond M.M.H.T., Rovers M.M., Hannink G., Hendriks C.T.M., van Heerbeek N. (2019). Septoplasty with or without concurrent turbinate surgery versus non-surgical management for nasal obstruction in adults with a deviated septum: A pragmatic, randomised controlled trial. Lancet.

[4] Carrie S., O’Hara J., Fouweather T., Homer T., Rousseau N., Rooshenas L., Bray A., Stocken D.D., Ternent L., Rennie K. (2023). Clinical effectiveness of septoplasty versus medical management for nasal airways obstruction: Multicentre, open label, randomised controlled trial. BMJ.

[5] Taha H.I., Elgendy M.S., Ezz M.R., Tolba K., El Safty M., Al Azzawi M.A.D.A., Katamesh B.E., Albazee E. (2025). Septoplasty versus non-surgical management for deviated nasal septum: A systematic review and meta-analysis of randomized controlled trials. Eur. Arch. Otorhinolaryngol..

[6] Sunnergren O., Alexandersson C., Broström A., Eliasson F., Jangard M., Lilja Y., Pakpour A., Sandelin K., Ahlström-Emanuelsson C. (2025). Clinical practice and outcome of septoplasty—A retrospective study of 11,714 surgeries in Sweden 2014–2023. Laryngoscope Investig. Otolaryngol..

[7] Bin Lajdam G., Alaryani K., Ghaddaf A.A., Aljabri A., Halawani A., Alshareef M., Algarni M., Al-Hakami H. (2022). Septoplasty versus septoplasty with turbinate reduction for nasal obstruction due to deviated nasal septum: A systematic review and meta-analysis. Rhinology.

[8] André R.F., Vuyk H.D., Ahmed A., Graamans K., Nolst Trenité G.J. (2009). Correlation between subjective and objective evaluation of the nasal airway. A systematic review of the highest level of evidence. Clin. Otolaryngol..

[9] Stewart M.G., Smith T.L. (2005). Objective versus subjective outcomes assessment in rhinology. Am. J. Rhinol..

[10] Lam D.J., James K.T., Weaver E.M. (2006). Comparison of anatomic, physiological, and subjective measures of the nasal airway. Am. J. Rhinol..

[11] Verhoeven S., Schmelzer B. (2016). Type and severity of septal deviation are not related with the degree of subjective nasal obstruction. Rhinology.

[12] Braithwaite I.T., Jones A., Doherty C.M., Sharma D.K. (2026). Computational fluid dynamics and virtual septoplasty in nasal airway obstruction: A narrative review. Cureus.

[13] Tricco A.C., Lillie E., Zarin W., O’Brien K.K., Colquhoun H., Levac D., Moher D., Peters M.D.J., Horsley T., Weeks L. (2018). PRISMA Extension for Scoping Reviews (PRISMA-ScR): Checklist and Explanation. Ann. Intern. Med..

[14] Hariri B.M., Rhee J.S., Garcia G.J.M. (2015). Identifying patients who may benefit from inferior turbinate reduction using computer simulations. Laryngoscope.

[15] Bahmanzadeh H., Abouali O., Faramarzi M., Ahmadi G. (2015). Numerical simulation of airflow and micro-particle deposition in human nasal airway pre- and post-virtual sphenoidotomy surgery. Comput. Biol. Med..

[16] Wofford M.R., Kimbell J.S., Frank-Ito D.O., Dhandha V., McKinney K.A., Fleischman G.M., Ebert C.S., Zanation A.M., Senior B.A. (2015). A computational study of functional endoscopic sinus surgery and maxillary sinus drug delivery. Rhinology.

[17] Simmen D., Sommer F., Briner H.R., Jones N., Kröger R., Hoffmann T.K., Lindemann J. (2015). The effect of “pyriform turbinoplasty” on nasal airflow using a virtual model. Rhinology.

[18] Lee K.B., Jeon Y.S., Chung S.K., Kim S.K. (2016). Effects of partial middle turbinectomy with varying resection volume and location on nasal functions and airflow characteristics by CFD. Comput. Biol. Med..

[19] Dayal A., Rhee J.S., Garcia G.J.M. (2016). Impact of middle versus inferior total turbinectomy on nasal aerodynamics. Otolaryngol. Head Neck Surg..

[20] Chung S.K., Kim D.W., Na Y. (2016). Numerical study on the effect of uncinectomy on airflow modification and ventilation characteristics of the maxillary sinus. Respir. Physiol. Neurobiol..

[21] Clipp R.B., Vicory J., Horvath S., Mitran S., Kimbell J.S., Rhee J.S., Enquobahrie A. (2018). An interactive, patient-specific virtual surgical planning system for upper airway obstruction treatments. Annu. Int. Conf. IEEE Eng. Med. Biol. Soc..

[22] Lee T.S., Goyal P., Li C., Zhao K. (2018). Computational fluid dynamics to evaluate the effectiveness of inferior turbinate reduction techniques to improve nasal airflow. JAMA Facial Plast. Surg..

[23] Sanmiguel-Rojas E., Burgos M.A., Esteban-Ortega F. (2018). Nasal surgery handled by CFD tools. Int. J. Numer. Method Biomed. Eng..

[24] Burgos M.A., Sanmiguel-Rojas E., Rodríguez R., Esteban-Ortega F. (2018). A CFD approach to understand nasoseptal perforations. Eur. Arch. Otorhinolaryngol..

[25] Burgos M.A., Sevilla García M.A., Sanmiguel-Rojas E., Del Pino C., Fernández Vélez C., Piqueras F., Esteban Ortega F. (2018). Virtual surgery for patients with nasal obstruction: Use of computational fluid dynamics (MeComLand^®^, Digbody^®^ & Noseland^®^) to document objective flow parameters and optimise surgical results. Acta Otorrinolaringol. Esp..

[26] Burgos M.A., Sanmiguel-Rojas E., Singh N., Esteban-Ortega F. (2018). DigBody^®^: A new 3D modeling tool for nasal virtual surgery. Comput. Biol. Med..

[27] Chung S.K., Byun S., Na Y. (2018). Investigation of flow characteristics in the maxillary sinus where polypoid changes develop. Comput. Biol. Med..

[28] Berger M., Pillei M., Mehrle A., Recheis W., Kral F., Kraxner M., Freysinger W. (2019). Predicting resection volumes within the nasal cavity to improve patients’ breathing. Proceedings of the Medical Imaging 2019: Computer-Aided Diagnosis, Proc. SPIE 10950, San Diego, CA, USA, 17–20 February 2019.

[29] Frank-Ito D.O., Kimbell J.S., Borojeni A.A.T., Garcia G.J.M., Rhee J.S. (2019). A hierarchical stepwise approach to evaluate nasal patency after virtual surgery for nasal airway obstruction. Clin. Biomech..

[30] Farzal Z., Del Signore A.G., Zanation A.M., Ebert C.S., Frank-Ito D., Kimbell J.S., Senior B.A. (2019). A computational fluid dynamics analysis of the effects of size and shape of anterior nasal septal perforations. Rhinology.

[31] Li L., London N.R., Zang H., Han D. (2020). Impact of posterior septum resection on nasal airflow pattern and warming function. Acta Otolaryngol..

[32] Xiong H., Cao H., Huang Y. (2020). An optimization method for surgical reduction of hypertrophied inferior turbinate. J. Biomech..

[33] Radulesco T., Meister L., Bouchet G., Varoquaux A., Giordano J., Dessi P., Perrier P., Michel J. (2020). Computational fluid dynamics and septal deviations—Virtual surgery can predict post-surgery results: A preliminary study including two patients. Clin. Otolaryngol..

[34] Siu J., Inthavong K., Shang Y., Vahaji S., Douglas R.G. (2020). Aerodynamic impact of total inferior turbinectomy versus inferior turbinoplasty—A computational fluid dynamics study. Rhinology.

[35] Moghaddam M.G., Garcia G.J.M., Frank-Ito D.O., Kimbell J.S., Rhee J.S. (2020). Virtual septoplasty: A method to predict surgical outcomes for patients with nasal airway obstruction. Int. J. Comput. Assist. Radiol. Surg..

[36] Saibene A.M., Felisati G., Pipolo C., Bulfamante A.M., Quadrio M., Covello V. (2020). Partial preservation of the inferior turbinate in endoscopic medial maxillectomy: A computational fluid dynamics study. Am. J. Rhinol. Allergy.

[37] Campbell D.A., Moghaddam M.G., Rhee J.S., Garcia G.J.M. (2021). Narrowed posterior nasal airway limits efficacy of anterior septoplasty. Facial Plast. Surg. Aesthet. Med..

[38] Senanayake P., Salati H., Wong E., Bradshaw K., Shang Y., Singh N., Inthavong K. (2021). The impact of nasal adhesions on airflow and mucosal cooling—A computational fluid dynamics analysis. Respir. Physiol. Neurobiol..

[39] Siu J., Inthavong K., Dong J., Shang Y., Douglas R.G. (2021). Nasal air conditioning following total inferior turbinectomy compared to inferior turbinoplasty—A computational fluid dynamics study. Clin. Biomech..

[40] Inthavong K., Shang Y., Del Gaudio J.M., Wise S.K., Edwards T.S., Bradshaw K., Wong E., Smith M., Singh N. (2021). Inhalation and deposition of spherical and pollen particles after middle turbinate resection in a human nasal cavity. Respir. Physiol. Neurobiol..

[41] Moniripiri M., Amjadimanesh H., Faramarzi M., Sadrizadeh S., Abouali O. (2021). Numerical simulation of unsteady airflow in a nasal cavity for various sizes of maxillary sinus opening in a virtual endoscopic surgery. Respir. Physiol. Neurobiol..

[42] Waldmann M., Rüttgers M., Lintermann A., Schröder W. (2022). Virtual surgeries of nasal cavities using a coupled lattice-Boltzmann–level-set approach. ASME J. Med. Diagn..

[43] Tao F., Feng Y., Sun B., Wang J., Chen X., Gong J. (2022). Septoplasty effect on the enhancement of airflow distribution and particle deposition in nasal cavity: A numerical study. Healthcare.

[44] Moshksayan K., Bahmanzadeh H., Faramarzi M., Sadrizadeh S., Ahmadi G., Abouali O. (2022). In-silico investigation of airflow and micro-particle deposition in human nasal airway pre- and post-virtual transnasal sphenoidotomy surgery. Comput. Methods Biomech. Biomed. Engin..

[45] Han S.C., Na Y., Won T.B. (2023). The effects of various maxillary sinus antrostomy techniques on modifying the ventilation and air-conditioning characteristics of the maxillary sinus: A numerical study. J. Rhinol..

[46] Senanayake P., Warfield-McAlpine P., Salati H., Bradshaw K., Wong E., Inthavong K., Singh N. (2023). The impact of adhesions on nasal airflow: A quantitative analysis using computational fluid dynamics. Am. J. Rhinol. Allergy.

[47] Khatri H., Salati H., Wong E., Bradshaw K., Inthavong K., Sacks R., Singh N. (2023). Modelling the effects of post-FESS middle turbinate synechiae on sinonasal physiology: A computational fluid dynamics study. Auris Nasus Larynx.

[48] Na Y., Kwon K.W., Jang Y.J. (2024). Impact of nasal septal perforation on the airflow and air-conditioning characteristics of the nasal cavity. Sci. Rep..

[49] Rüttgers M., Waldmann M., Vogt K., Ilgner J., Schröder W., Lintermann A. (2024). Automated surgery planning for an obstructed nose by combining computational fluid dynamics with reinforcement learning. Comput. Biol. Med..

[50] Macellari M., Schillaci A., Tanzini U., Trimarchi M., Quadrio M. (2024). An adjoint-based approach for the surgical correction of nasal septal deviations. Comput. Biol. Med..

[51] Güldiken Sarıkaya İ.N., Tekin A., Suda F., Çukurova Yilmaz Z.G., Özcan M. (2025). Modeling intra-sinus fluid movements and drainage through computational fluid dynamics before and after maxillary sinus augmentation: A simulation-based pilot study. J. Clin. Med..

[52] Odeh A., Wen R., Wu Z., Schneller A.R., Root Z.T., Hittle B., Wiet G.J., Otto B.A., Kelly K.M., Zhao K. (2025). Does total turbinectomy always lead to empty nose syndrome? A computational virtual surgery study. Laryngoscope.

[53] Pourmehran O., Psaltis A., Vreugde S., Zarei K., Shang Y., Inthavong K., Wormald P.J. (2025). Evaluating nebulization and nasal irrigation efficiency in post-operative chronic rhinosinusitis patients through computational fluid dynamics simulation. Comput. Methods Programs Biomed..

[54] Abbasi M., Amjadimanesh H., Sadrizadeh S., Abouali O. (2025). Experimental and numerical investigation of pressure distribution in human upper airway passage before and after maxillary sinus surgery. Respir. Physiol. Neurobiol..

