# Supplementary material for: Virtual Surgery and CFD in Rhinology: A Scoping Review
Source: J Clin Med. 2026 Oct 17;15(20):8069. doi: 10.3390/jcm15208069 (PMC13705948; doi:10.3390/jcm15208069)
Supplement: Supplementary file 1 [file jcm-15-08069-s001.zip › jcm-4578162-supplementary.pdf]

Supplementary Materials

Virtual surgery and CFD in rhinology: a scoping review

Table S1: Reports excluded after full-text assessment. Table S2: Complete search strategies for PubMed and Scopus. Table S3: PRISMA-ScR checklist.

Table S1. Reports excluded after full-text assessment (n = 4)

Reasons are those recorded in Figure 1 (“Not pertaining to the topic”, n = 4), with a short explanation of each in terms of the eligibility criteria in Section 2.2.

Report	Reason recorded in Figure 1	Explanation
Richards JP, Done AJ, Barber SR, Jain S, Son YJ, Chang EH. Virtual coach: the next tool in functional endoscopic sinus surgery education. Int Forum Allergy Rhinol 2020. doi:10.1002/alr.22452	Not pertaining to the topic	Simulator for training in endoscopic sinus surgery. No CFD analysis of airflow.
Hildebrandt T, Brüning JJ, Lamecker H, Zachow S, Heppt WJ, Schmidt N, Goubergrits L. Digital analysis of nasal airflow facilitating decision support in rhinosurgery. Facial Plast Surg 2019;35:3–8. doi:10.1055/s-0039-1677720	Not pertaining to the topic	Airflow analysis for decision support. No virtual surgical intervention was performed in the digital model.
Malik J, Thamboo A, Dholakia S, Borchard NA, McGhee S, Li C, Zhao K, Nayak JV. The cotton test redistributes nasal airflow in patients with empty nose syndrome. Int Forum Allergy Rhinol 2020;10(4):539–545. doi:10.1002/alr.22489	Not pertaining to the topic	No virtual surgical intervention (empty nose syndrome and the cotton test).
Balakin BV, Farbu E, Kosinski P. Aerodynamic evaluation of the empty nose syndrome by means of computational fluid dynamics. Comput Methods Biomech Biomed Eng 2017. doi:10.1080/10255842.2017.1385779	Not pertaining to the topic	CFD on actual pre- and postoperative CT scans. No virtual surgical intervention.

Three further reports could not be retrieved in full text (Figure 1). They are not listed here because PRISMA-ScR asks for reasons only for reports assessed in full text.

Table S2. Search strategies as run

Both databases were last searched in October 2025. Both searches were limited to publication years 2015–2025 and to English-language records. The search yielded 694 records (PubMed 349, Scopus 345).

Database	Query
PubMed (last search: October 2025)	("computational fluid dynamics"[Title/Abstract] OR "CFD"[Title/Abstract] OR "numerical simulation"[Title/Abstract] OR "airflow simulation"[Title/Abstract] OR "flow simulation"[Title/Abstract] OR "computational modeling"[Title/Abstract] OR "computational modelling"[Title/Abstract] OR "numerical model"[Title/Abstract]) AND (nasal OR nose OR sinonasal OR "nasal cavity" OR "nasal airway" OR "nasal obstruction" OR "nasal septum" OR septal OR "paranasal sinus" OR "paranasal sinuses" OR turbinate OR turbinates OR rhinology OR rhinologic OR rhinosinusitis OR "nasal valve" OR "empty nose") AND (surgery OR surgical OR virtual OR "surgical planning" OR "surgery planning" OR septoplasty OR turbinectomy OR turbinoplasty OR antrostomy OR maxillectomy OR FESS OR "sinus surgery" OR resection) [Limits applied: publication date 2015–2025; language English. In PubMed the [Title/Abstract] tag was applied to the first block only; the second and third blocks were entered without field tags.]
Scopus (last search: October 2025)	TITLE-ABS-KEY ("computational fluid dynamics" OR "CFD" OR "numerical simulation" OR "airflow simulation" OR "flow simulation" OR "computational modeling" OR "computational modelling" OR "numerical model") AND TITLE-ABS-KEY (nasal OR nose OR sinonasal OR "nasal cavity" OR "nasal airway" OR "nasal obstruction" OR "nasal septum" OR septal OR "paranasal sinus" OR "paranasal sinuses" OR turbinate OR turbinates OR rhinology OR rhinologic OR rhinosinusitis OR "nasal valve" OR "empty nose") AND TITLE-ABS-KEY (surgery OR surgical OR virtual OR "surgical planning" OR "surgery planning" OR septoplasty OR turbinectomy OR turbinoplasty OR antrostomy OR maxillectomy OR FESS OR "sinus surgery" OR resection) AND PUBYEAR > 2014 AND PUBYEAR < 2026 AND (LIMIT-TO (LANGUAGE , "English"))

Table S3. PRISMA-ScR checklist

Preferred Reporting Items for Systematic reviews and Meta-Analyses extension for Scoping Reviews (PRISMA-ScR) checklist [13]. Items are reported by section of the revised manuscript because page numbers change during production.

Section	Item	#	PRISMA-ScR checklist item	Reported in (revised manuscript)
TITLE	Title	1	Identify the report as a scoping review.	Title ("…: a scoping review")
ABSTRACT	Structured summary	2	Provide a structured summary that includes (as applicable): background, objectives, eligibility criteria, sources of evidence, charting methods, results, and conclusions that relate to the review questions and objectives.	Abstract: background and objective; methods (sources, date of search, eligibility criteria, screening, charting, registration); results; conclusions. Charting methods are described in full in Section 2.4.
INTRODUCTION	Rationale	3	Describe the rationale for the review in the context of what is already known. Explain why the review questions/objectives lend themselves to a scoping review approach.	Introduction, paragraphs 1–3 (including the difference from reference 12 and the reason for choosing a scoping review).
	Objectives	4	Provide an explicit statement of the questions and objectives being addressed with reference to their key elements (e.g., population or participants, concepts, and context) or other relevant key elements used to conceptualize the review questions and/or objectives.	Introduction, last paragraph: four review questions (applications, computational methods, validation, clinical outcomes). Population: human subjects or human-derived models; concept: CFD-based virtual surgery; context: rhinology (nose and paranasal sinuses).
METHODS	Protocol and registration	5	Indicate whether a review protocol exists; state if and where it can be accessed (e.g., a Web address); and if available, provide registration information, including the registration number.	Section 2, first paragraph: no registered or published protocol; plan agreed informally by the authors before the search. See also Section 4.5.
	Eligibility criteria	6	Specify characteristics of the sources of evidence used as eligibility criteria (e.g., years considered, language, and publication status), and provide a rationale.	Section 2.2 (population, age, applications, publication types, boundary studies, language). Rationale for the year 2015: Introduction, last paragraph.
	Information sources*	7	Describe all information sources in the search (e.g., databases with dates of coverage and contact with authors to identify additional sources), as well as the date the most recent search was executed.	Section 2.1 (PubMed and Scopus, 2015–2025, last search October 2025; reference lists not screened; no other supplementary search). Authors were not contacted for unpublished data; the authors of one report were contacted only to obtain its full text (Section 4.5).
	Search	8	Present the full electronic search strategy for at least 1 database, including any limits used, such that it could be repeated.	Section 2.1 (PubMed strategy and limits) and Table S2 (both databases, as run).
	Selection of sources of evidence†	9	State the process for selecting sources of evidence (i.e., screening and eligibility) included in the scoping review.	Section 2.3 and Figure 1.
	Data charting process‡	10	Describe the methods of charting data from the included sources of evidence (e.g., calibrated forms or forms that have been tested by the team before their use, and whether data charting was done independently or in duplicate) and any processes for obtaining and confirming data from investigators.	Section 2.4 (independent charting by two authors in separate spreadsheets, comparison of entries, disagreements resolved by a third author; entries checked against the full texts; no data were requested from investigators). Limitations: Section 4.5.
	Data items	11	List and define all variables for which data were sought and any assumptions and simplifications made.	Section 2.4 and the legends of Tables 1 and 2 (variables, definition of "Validation", "None" and "NR").
	Critical appraisal of individual sources of evidence§	12	If done, provide a rationale for conducting a critical appraisal of included sources of evidence; describe the methods used and how this information was used in any data synthesis (if appropriate).	Not done; the reason is given in Section 2.4 (the aim was to map the evidence) and Section 4.5.
	Synthesis of results	13	Describe the methods of handling and summarizing the data that were	Section 2.4 (grouping by anatomical region and type of virtual

Section	Item	#	PRISMA-ScR checklist item	Reported in (revised manuscript)
			charted.	intervention; narrative summary; no meta-analysis).
RESULTS	Selection of sources of evidence	14	Give numbers of sources of evidence screened, assessed for eligibility, and included in the review, with reasons for exclusions at each stage, ideally using a flow diagram.	Figure 1; Section 2.3. Reports excluded after full-text assessment and their reasons: Table S1.
	Characteristics of sources of evidence	15	For each source of evidence, present characteristics for which data were charted and provide the citations.	Section 3.1; Tables 1, 2 and 3.
	Critical appraisal within sources of evidence	16	If done, present data on critical appraisal of included sources of evidence (see item 12).	Not applicable (see item 12).
	Results of individual sources of evidence	17	For each included source of evidence, present the relevant data that were charted that relate to the review questions and objectives.	Table 1 and Table 2; Sections 3.2–3.7.
	Synthesis of results	18	Summarize and/or present the charting results as they relate to the review questions and objectives.	Sections 3.1–3.7 and Table 3.
DISCUSSION	Summary of evidence	19	Summarize the main results (including an overview of concepts, themes, and types of evidence available), link to the review questions and objectives, and consider the relevance to key groups.	Section 4 (Sections 4.1–4.4).
	Limitations	20	Discuss the limitations of the scoping review process.	Section 4.5.
	Conclusions	21	Provide a general interpretation of the results with respect to the review questions and objectives, as well as potential implications and/or next steps.	Section 5.
FUNDING	Funding	22	Describe sources of funding for the included sources of evidence, as well as sources of funding for the scoping review. Describe the role of the funders of the scoping review.	Funding statement (no external funding for this review; the article processing charge is funded by the authors' institution). The funding of the included studies was not charted (Section 2.4).

* Where sources of evidence are compiled from, such as bibliographic databases, social media platforms, and Web sites. † A more inclusive/heterogeneous term used to account for the different types of evidence or data sources that may be eligible in a scoping review as opposed to only studies. ‡ The frameworks by Arksey and O'Malley and Levac and colleagues and the JBI guidance refer to the process of data extraction in a scoping review as data charting. § The process of systematically examining research evidence to assess its validity, results, and relevance before using it to inform a decision. From: Tricco AC, Lillie E, Zarin W, O'Brien KK, Colquhoun H, Levac D, et al. PRISMA Extension for Scoping Reviews (PRISMA-ScR): Checklist and Explanation. Ann Intern Med 2018;169:467–473. doi:10.7326/M18-0850.
